# Engineered fibroblast growth factor 1 variants uncouple glucose-lowering effects from mitogenic activity with therapeutic potential for type 2 diabetes

**DOI:** 10.1186/s43556-025-00398-w

**Published:** 2026-01-11

**Authors:** Aleksandra A. Czyrek, Daniel Krowarsch, Szymon Sidor, Michal Janiszewski, Ewa Drzazga-Wilk, Katarzyna Bazydlo-Guzenda, Pawel Buda, Jerzy Pieczykolan, Natalia Porebska, Marta Minkiewicz, Pavel Krejci, Maciej Wieczorek, Jacek Otlewski, Malgorzata Zakrzewska

**Affiliations:** 1 Department of Protein Engineering, Faculty of Biotechnology, University of Wroclaw, Joliot-Curie 14a, Wroclaw, 50-383 Poland; 2 Department of Biology, Faculty of Medicine, Masaryk University, Kamenice 753/5, Brno, 62500 Czech Republic; 3 International Clinical Research Center, St. Anne’s University Hospital, Pekarska 53, Brno, 65691 Czech Republic; 4 Institute of Animal Physiology and Genetics of the CAS, Rumburska 89, Libechov, 27721 Czech Republic; 5 Celon Pharma S.A., R&D Centre, Marymoncka 15, Kazun Nowy, 05-152 Poland; 6 Department of Medical Biotechnology, Faculty of Biotechnology, University of Wroclaw, Joliot-Curie 14a, Wroclaw, 50-383 Poland

**Keywords:** Fibroblast growth factor 1, Reduced proliferative activity, Glucose-lowering properties, Type 2 diabetes, Glucose uptake, Thermodynamic stability

## Abstract

Fibroblast growth factor 1 (FGF1), a well-characterized member of the FGF family, effectively lowers blood glucose levels in animal models of type 2 diabetes by stimulating glucose uptake. However, its significant mitogenic potential poses a major challenge for clinical application. Here, we present engineered variants of FGF1 designed to dissociate its potent glucose-lowering effects from its undesired proliferative activity, aiming for a future therapeutic agent for type 2 diabetes. Through a series of rational mutations focused on modulating receptor binding and heparan interactions, coupled with enhanced thermodynamic stability, we developed two lead FGF1 variants. Comprehensive in vitro studies confirmed that these variants exhibit significantly reduced mitogenic potential across various cell types compared to wild-type FGF1. Specifically, one variant showed profound loss of proliferation due to disrupted FGFR binding, while the other displayed attenuated mitogenicity linked to decreased heparin affinity. Critically, both fully maintained potent glucose-lowering properties in *db/db* mice without inducing hypoglycemia or changes in body weight. Furthermore, these engineered proteins demonstrate superior thermodynamic stability and markedly improved pharmacokinetic profile, critical attributes for drug development. Our findings highlight a successful strategy to uncouple the therapeutic benefits of FGF1 from its mitogenic side effects, offering promising, stable, and safe protein-based drug candidates for type 2 diabetes treatment.

**Supplementary Information:**

The online version contains supplementary material available at 10.1186/s43556-025-00398-w.

## Introduction

Mammalian fibroblast growth factors (FGFs) are involved in numerous cellular processes, including proliferation, migration, angiogenesis, development, and the maintenance of energy homeostasis [1, 2]. The FGF superfamily comprises 22 genes encoding structurally related polypeptides, specifically 15 paracrine canonical FGFs (including FGF1–10, FGF16–18, FGF20 and FGF22), three endocrine FGFs (FGF19, FGF21 and FGF23), and four intracrine FGFs (FGF11–14) [1, 3]. FGFs exert their biological activities through binding to, dimerizing and activating specific single-pass transmembrane receptor tyrosine kinases (RTKs), known as FGF receptors (FGFR1-FGFR4) [4]. However, individual FGFs exhibit different specificity towards particular splicing variants of FGFRs [5]. Additionally, paracrine FGFs strongly interact with heparan sulfate proteoglycans (HSPGs), which stabilize the FGF-FGFR complex. While the precise mechanisms controlling FGFR signaling remain to be fully elucidated, it is known that the cellular response induced by FGF-FGFR interactions is spatially and temporally regulated and dependent on the complex's dimerization strength [6, 7].

For many years, only endocrine FGFs were considered crucial for energy homeostasis [2, 8]. However, recent studies demonstrate that canonical FGFs can also effectively induce glucose uptake [9–11]. Consequently, they are increasingly recognized as potential agents for type 2 diabetes (T2D), offering an advantage over current drugs like metformin by lacking their associated drawbacks [12–15]. FGF1, in particular, appears highly promising, exhibiting potent antidiabetic properties in mouse models. While the exact relationship between serum FGF1 levels and metabolic diseases is not fully understood [16–19], FGF1 is considered a critical transducer of PPARγ signaling, which is essential for the proper coupling of nutrient storage to adaptive adipose tissue remodeling [12]. Furthermore, FGF1 stimulate glucose uptake in adipocytes via an alternative pathway, independently of insulin [20].

Unfortunately, the therapeutic application of FGF1 protein carries inherent risks due to its mitogenic potential [21]. Therefore, numerous research groups are developing FGF1 variants or chimeras designed to possess reduced proliferative properties while retaining metabolic activity [6, 10, 22]. Despite these efforts, no FGF1 analogue capable of effectively lowering glucose levels and acting against diabetes has yet reached human clinical trials [23].

In this study, we described and characterized a series of engineered FGF1 variants with potential as blood glucose-lowering therapeutic proteins. To reduce the proliferative activity of FGF1, we designed a set of mutations that disrupt the interaction with FGFRs. In one of the final variants, we also incorporated a previously described mutation that reduces mitogenic potential of FGF1 by impairing heparin binding [24]. Furthermore, we utilized stabilizing mutations to enhance FGF1’s thermodynamic properties [25, 26], which could significantly benefit the protein's half-life, formulation and storage of the protein.

## Results

### Production of engineered FGF1 variants

**Fig. 1:**
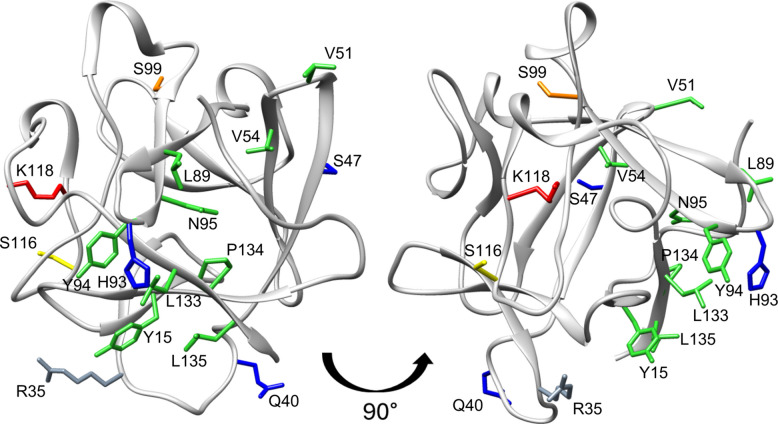
Three-dimensional structure of FGF1 (PDB code: 1RG8) indicating the location of mutated residues: involved in interaction with the FGF receptor (green) or heparin (Lys118, red); involved in binding to integrin αvβ3 (Arg35, grey); at the residue phosphorylated by PKCδ (Ser116, yellow), analogous to Ser117 in FGF2 (Ser99, orange); and substituted to increase protein stability (blue)

Based on the concept that prolonged exposure of cells to growth factor and maintenance of a stable FGF:FGFR complex are critical for proliferative activity, we rationally designed FGF1 mutants to reduce FGF1's affinity for its receptor and for heparin. We also incorporated mutations aimed at improving protein stability and disrupting other potential interactions. To reduce FGF1-FGFR binding, we introduced 14 novel point mutations to reduce FGF1’s affinity to FGFR1 primarily targeting van der Waals interactions, hydrophobicity, and side-chain properties, as guided by structural analyses (Fig. 1). Specifically, V51A, V54A, L89A, N95T, L133A and P134A substitutions were designed to shorten amino acid side chains, thereby potentially diminishing van der Waals contacts. Change in in amino acid residue hydrophobicity were explored with L89T, L133T, and P134S variants (increasing polarity to weaken of van der Waals interactions) and with Y94F and N95V muteins (increasing hydrophobicity to remove hydrogen bonds). Substitutions N95D, L135D, and L135R aimed to alter the side chain characteristic (Table 1).

**Table 1:** Engineered FGF1 variants and their mitogenic activity. To assess the mitogenic activity of FGF1 variants, serum-starved NIH 3T3 cells were stimulated with FGF1 mutants in the concentration range of 0.1–100 ng/mL in the presence of 10 U/mL heparin for 48 h. Data are presented as the mean ratio of the EC_50_ of mutants to the EC_50_ of wild-type (WT) FGF1 ± SEM, *n* = 3. Statistical significance: NS – non-significant, *p ≤ 0.05; **p ≤ 0.01 and ***p ≤ 0.001. The order of mutants within each group corresponds to decreasing mitogenic potential. ND—not determined

**FGF1 variant**		**EC**_**50**_** variant/EC**_**50**_** WT**	**SEM**	**significancy**		**reference**
**wild-type**		1	-	-		
**stable**						
	Q40P/S47I/H93G	1.37	0.58	NS		[25, 26]
**with disrupted FGFR interactions**						
	V51A	0.8	0.09	NS		
	Y94F	0.93	0.13	NS		
	P134A	0.96	0.15	NS		
	P134S	1.23	0.11	NS		
	L89T	1.53	0.27	NS		
	N95T	2.15	0.23	**		
	L135R	2.94	0.09	***		
	L133T	3.02	0.6	*		
	L89A	3.55	0.42	**		
	N95A	4.31	0.76	*		[27]
	L133A	4.41	0.04	***		
	Y94A	5.27	0.21	***		[27]
	Y15A	6.3	0.56	***		[27]
	N95V	9.81	0.45	***		
	V54A	16.29	4.97	*		
	N95D	97.12	3.12	***		
	L135D	279.11	5.87	***		
	Y94A/N95A	639.37	542.1	***		[27]
**with impaired heparin binding**						
	K118E	> 1000	ND	ND		[24]
**with impaired αvβ3 integrin binding**						
	R35E	2.68	0.11	***		[29]
**analogous to FGF2 S117A**						
	S99A	3.27	0.38	**		
**with mutated PKCδ phosphorylation site**						
	S116R	1.35	0.33	NS		[22, 31]
**multiple**						
	R35E/Q40P/S47I/H93G	1.15	0.51	NS		[30]
	Q40P/S47I/H93G/S99A	1.22	0.25	NS		
	Q40P/S47I/H93G/S99A/K118E	73.4	11.39	**		
	Q40P/S47I/H93G/L135D	98	10.62	***		

In addition, four previously described FGF1 variants with reduced affinity for FGFR, Y15A, Y94A, N95A and Y94A/N95A, were also included in the study [27]. To further modulate FGF1 activity, we targeted its affinity for heparin, recognizing that heparin significantly stabilizes the FGF:FGFR complex. For this, we introduced the K118E, which causes a charge change, affecting the hydrogen and van der Waals interactions network [24]. We also included the Ser116 to Arg substitution, previously shown to altered the protein's charge [22], and the S99A mutation, hypothesized to disrupt FGF1 interaction with CK2 based on FGF2 experimental data [28]. The Arg35 residue, known to be involved in integrin αvβ3 interaction was also analyzed [29, 30]. Single mutations were introduced by site-directed mutagenesis (Tables S1, S2, S3). Selected single mutations were subsequently combined in multiple mutants, which additional incorporated three substitutions (Q40P, S47I and H93G) known to increase protein stability [25], thereby ensuring desired thermodynamic properties (Table 1).

All designed mutant proteins were expressed in *E. coli* BL21(DE3)pLysS and successfully purified. The expression yield ranged from 20 to 160 mg of pure protein per liter of bacterial culture. Their high solubility allowed purification from supernatants obtained after bacterial cell disintegration and centrifugation. The proteins were purified using a Heparin-Sepharose column. For proteins with impaired heparin binding (i.e., those with the K118E mutation), an additional gel filtration chromatography step was necessary. The native conformation of all variants was confirmed by fluorescence spectroscopy (Fig. S1), showing no significant changes compared to wild-type FGF1. These results indicate successful production of FGF1 variants with targeted modifications and preserved native fold.

### Effect of point mutations on mitogenic activity of FGF1 in NIH 3T3 cells

The significant mitogenic activity of FGF1 remains a primary concern, potentially limiting its therapeutic application in type 2 diabetes (T2D) treatment. To mitigate this, we engineered point mutations aimed at disrupting the stability of the FGF1:FGFR complex, thereby reducing its proliferative potential. We first evaluated the mitogenic activity of these FGF1 variants using the NIH 3T3 cell model. Serum-starved NIH 3T3 cells were stimulated with mutants in the concentration range of 0.1–100 ng/mL in the presence of 10 U/mL heparin for 48 h. The addition of heparin to the experiment made the cells more sensitive to stimulation with FGF1 proteins, resulting in increased proliferation. Using GraFit software, we calculated the EC_50_ (half maximal effective concentration) for each variant (Table 1). Our analysis revealed that variants: Y94A, Y15A, N95V, V54A, N95D, Y94A/N95A, L135D, Q40P/S47I/H93G/L135D, K118E, and Q40P/S47I/H93G/S99A/K118E showed significantly reduced mitogenic activity, with EC_50_ value at least fivefold higher than that of the wild-type protein). Consistent with previous findings, the R35E variant, known for its impaired integrin binding [29], displayed reduced proliferative activity compared to wild-type FGF1. However, the introduction of three stabilizing mutations restored its activity to wild-type levels [30]. The mutation of Ser116, which is required for FGF1 phosphorylation by PKCδ [31], to Arg did not affect FGF1 mitogenicity.

### Biological activity of FGF1 mutants

**Fig. 2:**
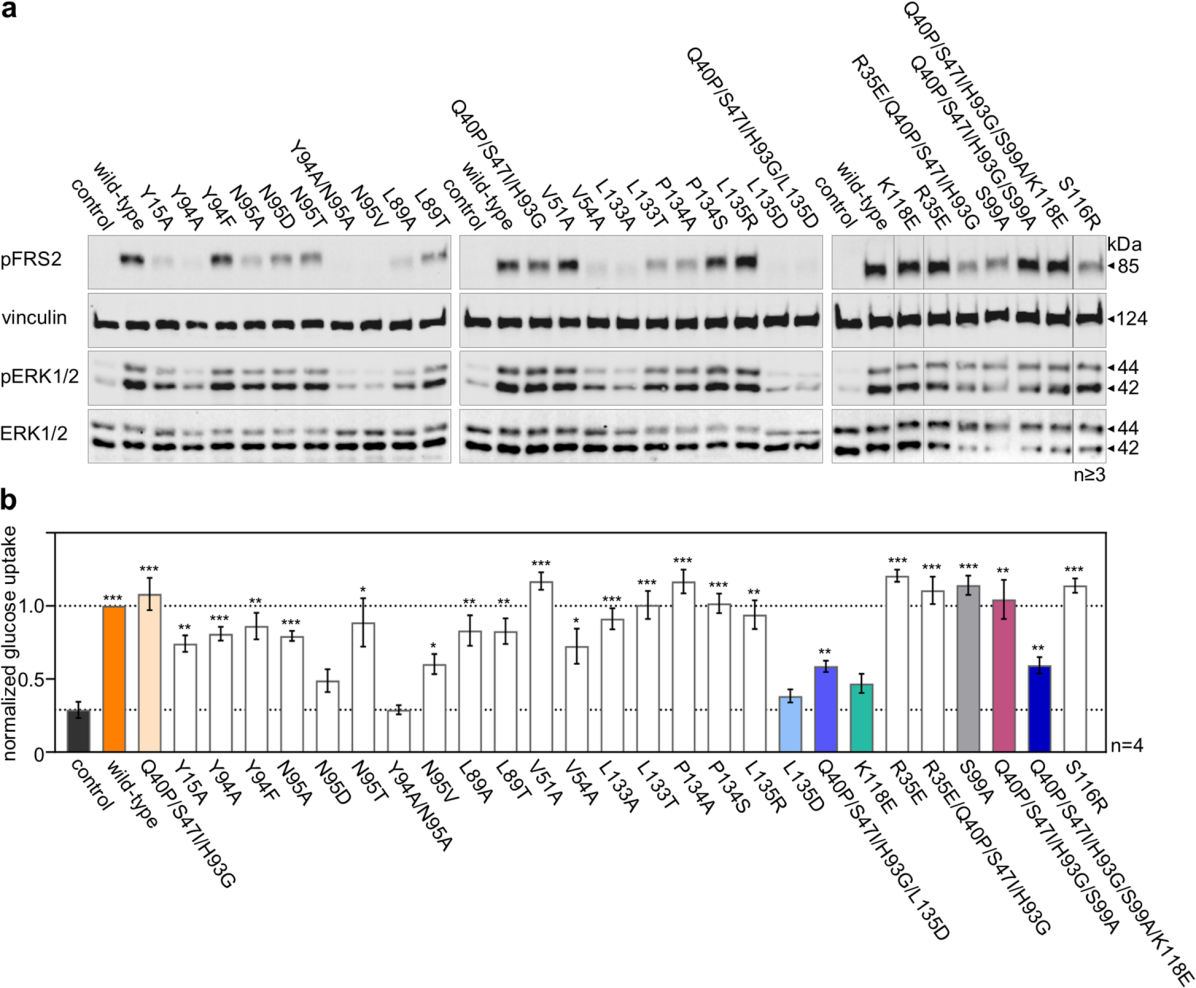
Effect of introducing point mutations on the biological activity of FGF1. **a** Serum-starved NIH 3T3 cells were treated with 10 ng/mL FGF1 variants for 15 min in the presence of heparin (10 U/mL). Activation of the downstream cascade was detected by immunoblotting using the following antibodies: anti-phospho-FRS2 (pFRS2) and anti-phospho-ERK1/2 (pERK1/2). Anti-ERK1/2 and anti-vinculin antibodies were used to confirm equal loading. Representative results are shown (n ≥ 3). The vertical lines in the last WB panel show the deleted wells. The original membranes, together with the method of trimming, are presented in Fig. S2. Densitometric analysis of pERK/ERK is presented in Fig. S3. **b** Effect of 20-h FGF1 variants stimulation (20 ng/mL) in the presence of 10 U/mL heparin on glucose uptake by 3T3-L1 adipocytes. Data are presented as mean ± SEM, *n* = 4. Statistical significance: *p ≤ 0.05; **p ≤ 0.01 and ***p ≤ 0.001

Beyond its mitogenic effect, FGF1 is recognized for its critical role in inducing other cellular activities, including migration, anti-apoptotic processes, and glucose uptake, all mediated by FGFR-dependent signaling pathways [10]. Having identified variants with attenuated mitogenic potential, we proceeded to investigate their broader biological effects, specifically their capacity to activate downstream signaling pathways, particularly the MAPK cascade (assessed via ERK1/2 phosphorylation), and stimulate glucose uptake. To examine the impact of introduced mutations on signaling, serum-starved NIH 3T3 cells were treated with 10 ng/mL of FGF1 variants in the presence of heparin. Activation of the downstream cascade was subsequently detected by Western blot analysis, focusing on FRS2 and ERK1/2 phosphorylation (Fig. 2a, Fig. S3). While several mutants showed a slight reduction in MAPK activation, only the Y94A/N95A, N95V, L135D, and Q40P/S47I/H93G/L135D variants displayed no discernible downstream signaling activity at the level of untreated cells when tested at 10 ng/mL.

FGF1-induced glucose uptake was assessed in 3T3-L1 adipocytes following 20 h stimulation with 20 ng/mL of FGF1 variants in the presence of heparin in 3T3-L1 adipocytes (Fig. 2b). Notably, only the Y94A/N95A, L135D, N95D, and K118E variants failed to stimulate glucose metabolism in these adipocytes. These results indicate that despite the lack of significant activation of FRS2 and ERK1/2 by certain FGF1 variants at a concentration of 10 ng/mL, including Q40P/S47I/H93G/L135D, they were still capable of maintaining metabolic activity. To explain the observed differences between downstream signaling and glucose uptake activity for the Q40P/S47I/H93G/L135D variant, we re-evaluated its signaling response at a higher concentration of 50 ng/mL. Under these conditions, it induced ERK1/2 activation in NIH 3T3 cells comparable to that of the wild-type protein (Fig. S4), which may explain the maintenance of metabolic activity. Even very weak activation of FGFR appears to be sufficient to elicit an antidiabetic effect, which is consistent with previous reports [6]. At this point, we have identified several mutants that show impaired proliferative activity while effectively stimulating glucose uptake in an in vitro adipocyte model.

### Analysis of binding of FGF1 variants with reduced proliferative potential to the FGF receptor

**Fig. 3:**
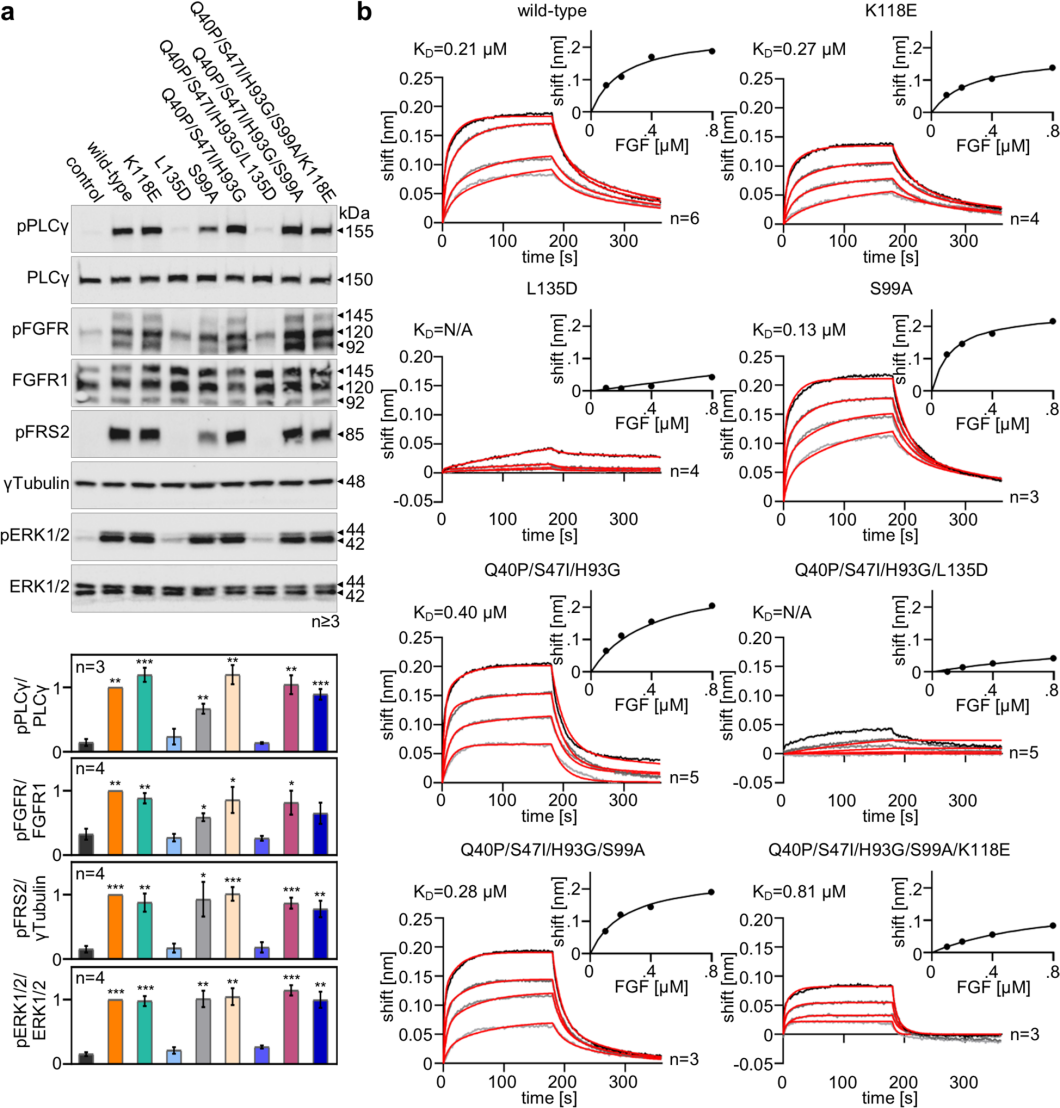
Impaired activation of signaling pathways by FGF1 variants due to reduced affinity for the FGFR1 (IIIc) receptor. **a** Serum-starved NIH 3T3 cells were stimulated with 10 ng/mL FGF1 variants in the presence of heparin (10 U/mL) for 15 min, and activation of downstream signaling cascades was detected by immunoblotting using the following antibodies: anti-phospho-FGFR (pFGFR), anti-phospho-PLCγ (pPLCγ), anti-phosphoFRS2 (pFRS2), anti-phospho-ERK1/2 (pERK1/2). Anti-ERK1/2, anti-FGFR1, anti-PLCγ and anti-γTubulin antibodies were used to confirm equal loading. Representative results are shown. Densitometric analysis is presented as mean ± SEM, *n* = 3/4. Statistical significance: *p ≤ 0.05; **p ≤ 0.01 and ***p ≤ 0.001. **b** BLI analysis of the affinity of FGF1 variants for FGFR1-Fc (IIIc isoform). FGFR1-Fc was immobilized on a Protein A sensor and its interactions (association and dissociation) with selected FGF1 mutants were analyzed in the concentration range of 100–800 nM. Curves obtained by global fitting are marked in red. Representative results are shown (n ≥ 3). The equilibrium dissociation constant (K_D_) was calculated from the saturation binding curve

Given the lack of clear evidence identifying o which FGFR-dependent pathway is crucial for the metabolic effect, we evaluated the phosphorylation of key proteins within the FGFR-dependent cascade (pFGFR, pPLCγ, pFRS2, pERK1/2) (Fig. 3a). Using selected FGF1 variants that demonstrated reduced mitogenicity and preserved metabolic activity, we stimulated serum-starved NIH 3T3 cells and analyzed downstream signaling cascades. We observed no significant differences in FGFR, PLCγ, FRS2 and ERK1/2 phosphorylation induced by variants with S99A or K118E mutations relative to wild-type FGF1 and the Q40P/S47I/H93G variant (Fig. 3a). In contrast, variants containing the L135D mutation exhibited a complete impairment in the activation of the signaling cascades at a concentration of 10 ng/mL, although the Q40P/S47I/H93G/L135D variant retained partial antidiabetic activity (Fig. 2a-b). To confirm that the impaired properties of the FGF1 variants are indeed due to disrupted binding to the receptor, we assessed their affinity for FGFR1 (IIIc) using biolayer interferometry (Fig. 3b). For this purpose, FGFR1 (IIIc)-Fc was immobilized on a Protein A sensor, and associations and dissociations of selected FGF1 variants were analyzed. Equilibrium dissociation constants (K_D_) were calculated from the saturation binding curve (steady-state). The data obtained showed virtually no binding for variants with the L135D mutation, strongly indicating that Leu135 is crucial for ligand-receptor interaction and subsequent activation of downstream pathways. The combination of K118E and S99A mutations (present in the Q40P/S47I/H93G/S99A/K118E mutant) mildly attenuated the binding of this mutein to the receptor. However, this attenuated binding did not compromise the activation of signaling proteins in FGFR-dependent pathways (Fig. 3a). Our results therefore confirm that the reduced mitogenic potential of Q40P/S47I/H93G/L135D is attributable to its highly disturbed binding to the FGF receptor, whereas for Q40P/S47I/H93G/S99A/K118E, it rather stems from its reduced affinity to heparin.

### The stability of FGF1 variants affects cell fate

**Fig. 4:**
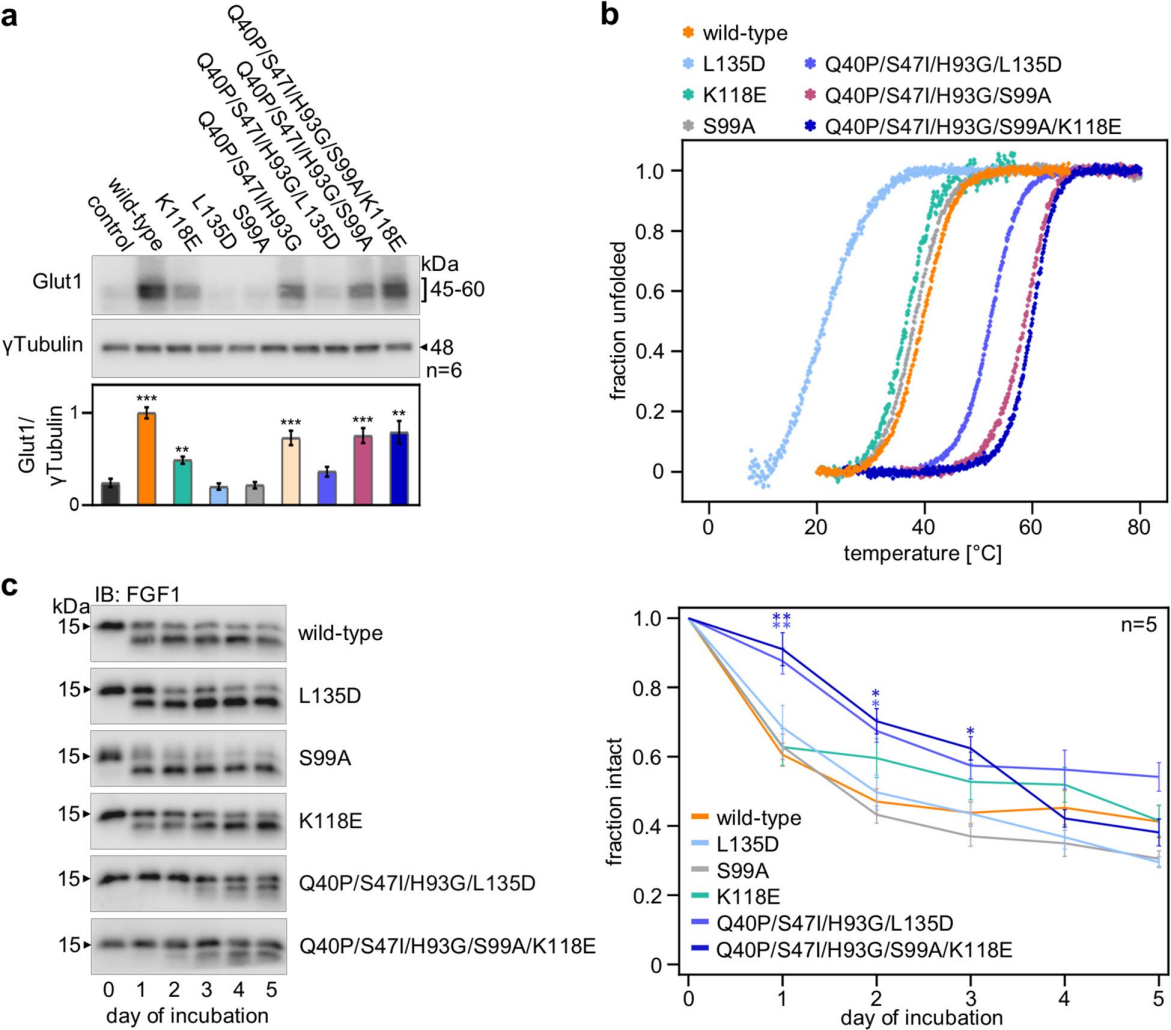
Effect of introduced mutations on the long-term activity of FGF1. **a** Induction of Glut1 expression after stimulation with FGF1 variants. 3T3-L1 cells were treated with 100 ng/mL FGF1 muteins in the presence of 10 U/mL heparin for 24 h, followed by WB analysis with anti-Glut1 and anti-γTubulin antibodies, data are presented as mean ± SEM, *n* = 6. Statistical significance: *p ≤ 0.05; **p ≤ 0.01 and ***p ≤ 0.001. **b** Normalized thermal denaturation curves of FGF1 variants monitored by ellipticity changes (λ = 227 nm). **c** Degradation of FGF1 variants in the presence of adipocytes. Serum-starved 3T3-L1 adipocytes were incubated with 1 µg/mL FGF1 variants. The progress of proteolysis on subsequent days was monitored by immunoblotting with anti-FGF1 antibody. Representative results are shown (*n* = 5). Densitometric analysis of proteolysis of FGF1 variants is presented as the ratio the intensity of the upper band to the intensity of the whole amount of protein. Mean ± SEM are shown, *n* = 5. Statistical significance: *p ≤ 0.05; **p ≤ 0.01 and ***p ≤ 0.001

FGF1 activates transcription factors, leading to the expression of glucose transporter 1 (Glut1), the primary transporter regulating basal glucose transport to adipocytes [20]. Interestingly, certain FGF1 muteins (K118E and S99A), which activated FGFR-dependent signaling pathways at a level comparable to the wild-type protein (Fig. 3a), showed weaker (K118E) or no (S99A) induction of Glut1 expression (Fig. 4a). We hypothesized that this discrepancy might stem from impaired stability, as even a single mutation can significantly alter a protein's thermodynamic parameters. Furthermore, verifying denaturation parameters is crucial due to the importance of protein stability in therapeutic applications. Protein-based drugs must possess sufficient stability to withstand formulation processes and to remain active until reaching target tissues. Therefore, the denaturation parameters of selected FGF1 variants were determined. Protein denaturation temperatures were analyzed in 25 mM phosphate buffer, pH 7.3, in the presence of 0.7 M GdmCl, which prevented protein aggregation and provided a two-state unfolding process for the FGF1 variants. Protein concentration during measurements was 2 µM, and denaturation was carried out with a temperature change at a rate of 0.25 °C/min. Unfolding was monitored by measuring fluorescence emission at λ = 353 nm (with λ = 280 nm excitation) and ellipticity at λ = 227 nm. Denaturation parameters, i.e. denaturation temperature (T_den_) and denaturation enthalpy change (∆H_den_), were calculated in PeakFit software assuming a two-state model of protein denaturation (Table S1).

Muteins S99A and K118E displayed slightly reduced T_den_ values relative to wild-type FGF1 (by 2.2 °C and 2.8 °C, respectively). These mutations were subsequently combined into a multiple variant alongside three known stabilizing mutations (Q40P, S47I, H93G). The Q40P/S47I/H93G/S99A/K118E variant exhibited significantly higher stability than the wild-type protein, with its T_den_ increasing to approximately 60 °C (Table S1, Fig. 4b). Mutation of Leu135 to Asp dramatically reduced the denaturation temperature and enthalpy of the protein. The introduction of three stabilizing mutations restored and even significantly improved the stability of the L135D mutein (the denaturation temperature of the Q40P/S47I/H93G/L135D variant was over 30 °C higher compared to the single L135D mutant). However, only Q40P/S47I/H93G/S99A/K118E variant effectively recovered the ability to stimulate Glut1 expression, whereas the Q40P/S47I/H93G/L135D variant, despite being more stable than the wild-type protein, failed to evoke this cellular response (Fig. 4a).

The improved thermodynamic parameters could manifest as enhanced protein properties, such as reduced aggregation or increased conformational stability, which translates into greater resistance to proteolytic degradation. Proteases present in the cell culture medium may be a major factor contributing to the time-dependent decrease in FGF1 activity. To assess the proteolytic stability of the obtained variants, we performed a proteolytic stability assay in the medium in the presence of 3T3-L1 adipocytes. For the stable muteins, Q40P/S47I/H93G/L135D and Q40P/S47I/H93G/S99A/K118E, degradation was reduced compared to the wild-type protein, with the variants persisting in the medium until day 2 and 3, respectively (Fig. 4c).

### Verification of the proliferative activity of selected FGF1 variants in different cell types

**Fig. 5:**
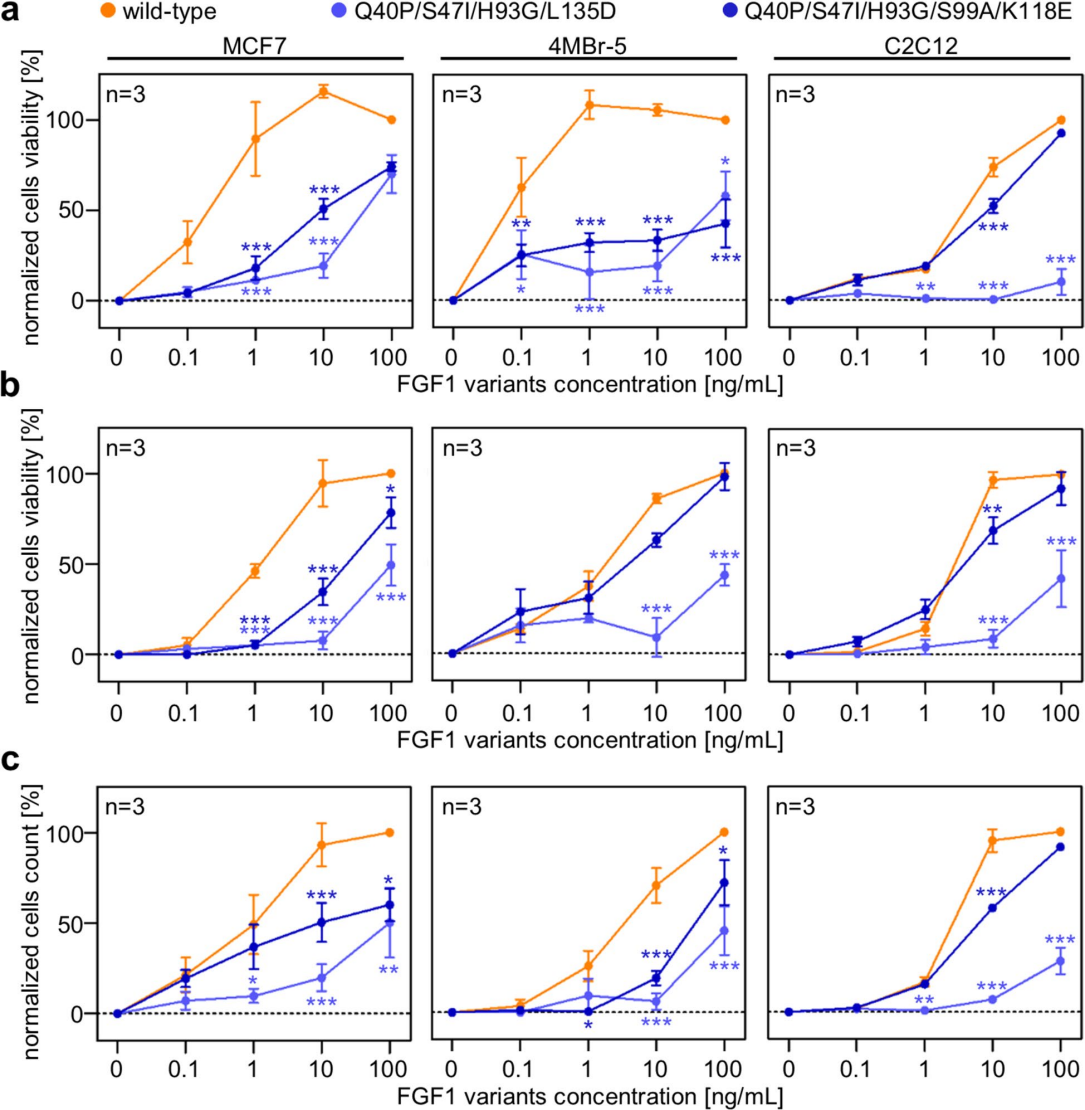
Mitogenic activity of selected FGF1 variants in different cell types. Mitogenic activity of selected FGF1 variants was assessed by stimulating serum-starved C2C12, MCF7 or 4MBr-5 cells with FGF1 mutants in the concentration range 0.1–100 ng/ml in the presence of 10 U/ml heparin for 48 h, 72 h or 96 h, respectively. Cell viability was assessed by PrestoBlue cell viability assay **a** or by CellTiter-Fluor cell viability assay **b**. Cell number was determined by counting NucBlue-stained nuclei using an Opera Phenix Plus High-Content Screening System **c**. Data are presented as mean ± SEM, *n* = 3. Multiple t-test; statistical significance: *p ≤ 0.05; **p ≤ 0.01 and ***p ≤ 0.001

Since FGF1 is a pan-activator of all FGFRs, we investigated the mitogenicity of selected variants – a property that represents a significant challenge for their therapeutic application. We utilized cell lines expressing not only FGFR1c but also other FGFRs, and exhibiting varying levels of heparan sulfate proteoglycans (HSPGs). To confirm their safety in terms of mitogenic activity, we evaluated the proliferative effects of the two most promising FGF1 variants, Q40P/S47I/H93G/L135D and Q40P/S47I/H93G/S99A/K118E, in mouse myoblasts (C2C12) [32], human mammary epithelial cells (MCF7) [33], and the rhesus monkey bronchial epithelial cell line (4MBr-5) [34]. We employed three distinct, yet complementary, cell viability assays: metabolic activity assay (PrestoBlue) (Fig. 5a), the membrane-integrity–dependent CellTiter-Fluor viability test (Fig. 5b), and direct cell counting (Fig. 5c).

In both MCF7 and 4MBR-5 cells, we consistently observed that the proliferation induced by the Q40P/S47I/H93G/L135D and Q40P/S47I/H93G/S99A/K118E variants was reduced compared to the wild-type protein. Notably, in C2C12 cells, the Q40P/S47I/H93G/S99A/K118E variant exhibited a proliferation profile comparable to that of the wild-type. This behavior is likely attributable to the molecular characteristics of the Q40P/S47I/H93G/S99A/K118E variant. Although it displays decreased heparin-binding affinity, resulting in a less stable ligand:receptor complex, C2C12 cells, even in their undifferentiated state, express a broad repertoire of heparan sulfate proteoglycans (e.g., syndecan-3, syndecan-4, glypican, and perlecan) [35]. The presence of these diverse HSPGs may compensate for the variant’s reduced affinity and facilitate the formation of a functionally stable signaling complex.

Across all three proliferation assays, wild-type FGF1 exhibited the highest mitogenic potency in MCF7, 4MBr-5, and C2C12 cells, as reflected by consistently low EC₅₀ values (Table S2). The Q40P/S47I/H93G/L135D variant showed a profound loss of mitogenic activity in all cell types, with EC₅₀ values increased by one to two orders of magnitude relative to wild-type. This indicates that the L135D substitution markedly impairs FGFR-mediated proliferative signaling even when the FGF1 protein is stabilized by additional mutations. The Q40P/S47I/H93G/S99A/K118E variant displayed an intermediate phenotype, with reduced potency compared to wild-type but substantially greater activity than the L135D-containing variant. Notably, the extent of this reduction was cell-type dependent, with MCF7 and 4MBr-5 cells showing a more pronounced decrease than C2C12 cells. The consistent trend observed for the variants across all assays underscores the robustness of these observations and demonstrating that specific substitutions can selectively attenuate the mitogenic activity of FGF1 (Fig. 5 and Table S2).

### In vivo characterization of the metabolic efficacy and pharmacokinetics of selected FGF1 variants

To evaluate the glucose lowering effect of selected FGF1 mutants in vivo, we utilized *db/db* mice, a model characterized by a mutation in the long isoform gene of the leptin receptor (*Lepr*). The initial blood glucose levels in these *db/db* mice ranged between 250 and 600 mg/dl. Blood sugar levels were monitored for 168 h using Accu-Chek®Performa. The purity of all protein variants used for in vivo testing exceeded 95% (Fig. S5). Animals received a single subcutaneous dose of the muteins at 1 mg/kg.

**Fig. 6:**
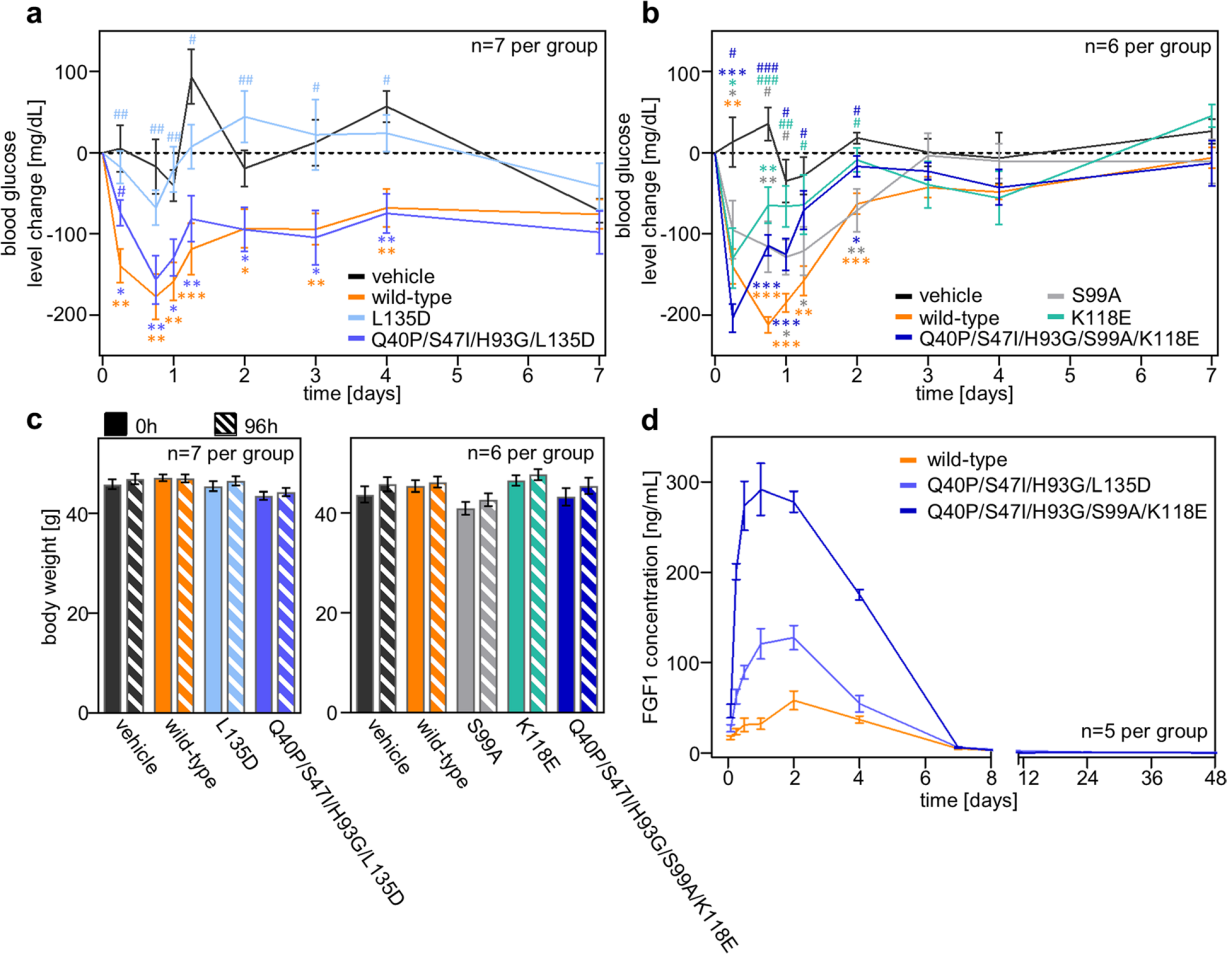
Metabolic activity of FGF1 variants in vivo. **a**, **b** Change in blood glucose levels in *db/db* mice after a single administration of FGF1 variants (measurements at 0, 6, 18, 24, 30, 48, 72, 96 and 168 h after protein administration). FGF1 variants were administered at a dose of 1 mg/kg body weight. Data were normalized to glucose levels before protein administration and presented as mean ± SEM, *n* = 7/6. Statistical significance *p ≤ 0.05; **p ≤ 0.01 and ***p ≤ 0.001; (*) indicates comparison with vehicle; (#) indicates comparison with wild-type protein. **c** Analysis of mouse body weight 96 h after administration. **d** Pharmacokinetics of FGF1 variants after a single subcutaneous administration in Wistar Han rats. FGF1 variants were administered subcutaneously at a dose 0.5 mg/kg, and blood samples were collected before injection and at 5 min, 15 min, 30 min and 1, 2, 4, 7, 12, 24, 48 h after injection and protein levels were analyzed by ELISA. Data are presented as mean ± SEM, *n* = 5

The maximum reduction in blood glucose was observed between 18 and 24 h after subcutaneous administration. The hypoglycemic effect induced by the wild-type protein persisted from 48 to 168 h (Fig. 6a-b). The L135D mutein, designed to lower FGF1's affinity for FGFR1, showed no metabolic activity. This lack of activity was attributed to the extremely low stability of the protein; notably, the subsequent introduction of stabilizing substitutions successfully restored the normoglycemic capacity of the L135D variant to a level comparable to that observed for the wild-type FGF1 protein.

The S99A mutant exhibited metabolic activity similar to that of the wild-type. However, the introduction of the K118E mutation into a stable S99A-containing variant (Q40P/S47I/H93G/S99A) resulted in reduced glucose-lowering activity, with an effect weaker than that of the wild-type protein. A similar curve profile was observed for the single K118E mutant variant. Furthermore, supplementary material includes box-and-whisker plots depicting the distribution and variance of glucose-lowering responses in *db/db* mice, thereby providing a comprehensive representation of inter-animal variability for each protein variant (Fig. S6). Importantly, none of the analyzed FGF1 variants induced the hypoglycemia often observed with many current antidiabetic drugs. Additionally, no significant changes in animal body weight were observed 96 h after protein administration (Fig. 6c).

As anticipated, the introduction of mutations designed to stabilize the protein and lower its affinity for heparin or FGFR resulted in an improved pharmacokinetic profile of FGF1 variants in Wistar Han rats (Fig. 6d, Table S3). Both tested variants, Q40P/S47I/H93G/L135D and Q40P/S47I/H93G/S99A/K118E, persisted in the body at significantly higher concentrations compared to the wild-type protein, up to 7 h post-administration. Their respective area under the curve (AUC) values were 533.1 ± 40.3 and 1261.8 ± 49, substantially greater than the 277.2 ± 21.5 observed for wild-type FGF1. Furthermore, significantly higher maximum concentrations (C_max_) were observed for both mutants, (141.4 ± 15.3 ng/mL for Q40P/S47I/H93G/L135D and 303.6 ± 24.6 ng/mL for Q40P/S47I/H93G/S99A/K118E) contrasting with 59.1 ± 10 ng/mL for wild-type FGF1. Times of maximum drug concentration (T_max_) did not differ significantly between the variants (Table S3). Our findings demonstrate that both engineered FGF1 variants effectively lower glucose levels in *db/db* mice and exhibit a superior pharmacokinetic profile.

## Discussion

For many years, only endocrine proteins from the FGF family were the subject of interest in the area of maintaining the body's metabolic homeostasis and developing methods for treating metabolic diseases, such as diabetes. However, most therapeutic attempts proved ineffective or led to significant side effects, including potassium and calcium disturbances, weight gain, or gastrointestinal problems [11, 36–38]. Over the past decade, FGF1, a canonical member of the fibroblast growth factor family, has emerged as a potent regulator of blood glucose levels [6, 9, 10, 22]. Studies in animal models of diabetes have demonstrated a significant advantage of FGF1 in glucose lowering: a lack of side effects, including those affecting the immune system. Nevertheless, FGF1's natural involvement in cell migration, differentiation, growth, and survival raises concerns that its therapeutic administration could disrupt natural homeostasis, potentially promoting the development of diseases coexisting with T2D, such as cancer [21]. This challenge necessitates the development of FGF1 variants that induce glucose uptake without proliferation activity.

To achieve these properties, we investigated a range of mutations designed to interfere with FGF1's interaction with FGFR, alongside a mutation known to reduce heparin affinity [24]. Recognizing the importance of protein stability for therapeutic agents, we additionally introduced stabilizing mutations that enhance FGF1's thermodynamic properties [25, 26]. Improved stability is highly beneficial for protein half-life, formulation, and storage [39]. Furthermore, we examined mutations at the Ser99, residue, implicated in FGF2 in proliferation [28]; Arg35, involved in integrin αvβ3 interaction [29, 30]; and Ser116, phosphorylated by PKCδ [22, 31]. From our extensive panel of mutants, we selected two multiple variants: Q40P/S47I/H93G/L135D and Q40P/S47I/H93G/S99A/K118E. These variants uniquely combine enhanced stability with significantly inhibited proliferative activity, while crucially retaining potent antidiabetic effects. This achievement was accomplished through two different design strategies: directly disrupting the interaction of FGF1 with the FGF receptor or weakening its binding to heparin.

Among the 19 variants engineered for potentially reduced FGFR affinity, eight exhibited a significant reduction in proliferative activity, with five showing a remarkable > tenfold reduction in NIH 3T3 cells. Variants with the most attenuated mitogenicity also displayed impaired activation of signaling pathways, which correlated with reduced in vitro metabolic activity (glucose uptake). These findings align with previous reports [6, 10, 22]. However, unlike the R35E variant, the introduction of stabilizing mutations into the L135D variant did not restore its mitogenic activity. Bioinformatics analysis of the FGF1:FGFR1:heparin using Ligplot software suggests that Leu135 is crucial not only for receptor binding, forming potential van der Waals interactions with four FGFR1 residues (Ala168, Asp247, Leu166, Val249) within a 5 Å radius, but also for maintaining the rigid structure of the FGF1 C-terminus via intramolecular interactions (Fig. S7). This indicates a dual role for Leu135 in both receptor interaction and protein structural integrity. The FGF1:FGFR1:heparin complex showed six potential hydrogen bonds involving Lys118, including three bonds to heparin that stabilize the FGF1:FGFR1 complex (Fig. S8) [40, 41]. Interestingly, variants containing the K118E mutation, despite exhibiting reduced mitogenicity and in vitro glucose uptake, maintained robust phosphorylation of FGFR-dependent signaling pathway proteins. This discrepancy might be explained by the rapid, short-term nature of the signaling response. Both selected multiple variants, Q40P/S47I/H93G/L135D and Q40P/S47I/H93G/S99A/K118E, successfully met our initial objectives by demonstrating highly impaired mitogenicity and at least partial activation of glucose uptake in 3T3-L1 adipocytes.

The Q40P/S47I/H93G/S99A/K118E variant characterized by reduced heparin affinity, exhibited attenuated proliferative activity, preserved ability to activate signaling cascades, as well as metabolic activity both in vitro (glucose uptake and increased expression of Glut1 protein) and in vivo (reduced glucose levels in *db/db* mice). These observations support the hypothesis that a weak FGF1:FGFR1 complex is sufficient for metabolic effect, whereas a strong, often heparin-stabilized complex, is essential for cell proliferation [6, 23, 24, 42]. The Q40P/S47I/H93G/L135D variant proved even more compelling due to its complete lack of proliferative activity, its ability to induce Glut1 expression, and its maintained in vivo metabolic properties at wild-type levels. Furthermore, the pharmacokinetic profile after subcutaneous administration were significantly improved for both selected variants compared to the wild-type protein. These collective properties position our newly engineered variants as promising candidates for a biological drug.

Our engineered proteins appear to possess superior properties compared to previously developed FGF1 variants for T2D therapy. For instance, the S116R FGF1 variant, enhanced for stability by Xia et al*.* [22], showed only a modest reduction in mitogenicity, whereas our variants achieved a much lower proliferative response, validated across multiple cell models (C2C12, MCF7, and 4MBr-5). Moreover, our heparin-binding–attenuated variant (Q40P/S47I/H93G/S99A/K118E) exhibits distinct behavior from the ΔHBS variants reported by Huang et al*.* [6]. While both approaches weaken the FGF1:FGFR1:HS complex, our variant uniquely maintains strong FGFR phosphorylation and elevated Glut1 expression, indicating a more selective suppression of mitogenic signaling while preserving durable metabolic effects. In contrast, chimeric FGF1ΔHBS protein with the C-terminal region of endocrine FGF21 (FGF1ΔHBS-FGF21C-tail) actually diminished its glucose-lowering efficacy in *db/db* mice [43]. Crucially, the mitogenic properties of our variants were evaluated across a broader range of cellular models than previously published studies. Despite FGF1's antidiabetic properties being described over a decade ago, no FGF1 variant has yet progressed to clinical evaluation for T2D [23].

The precise molecular mechanism regulating FGF1-induced glucose uptake remain to be fully elucidated. Our results for the Q40P/S47I/H93G/L135D variant offer insights into the FGF1-induced normoglycemia mechanism. The observed glucose lowering effect in the absence of Glut1 expression after Q40P/S47I/H93G/L135D stimulation suggests that the activation of either Glut4 translocation or Glut1 expression alone may be sufficient to achieve a normoglycemic outcome in vivo. In this context, both developed variants (Q40P/S47I/H93G/L135D and Q40P/S47I/H93G/S99A/K118E) show significant promise in circumventing the insulin resistance challenges prevalent in most diabetic patients.

Our findings for Q40P/S47I/H93G/S99A/K118E and Q40P/S47I/H93G/L135D strongly support the hypothesis by Huang et al*.* [6] that a relatively weak FGFR:FGF1 interaction is sufficient to trigger metabolic signaling, whereas robust receptor activation and dimerization are required for mitogenic responses. The strength and stability of the ligand:receptor complex are pivotal in determining these divergent outcomes. In this study, we deliberately attenuated receptor activation using two complementary strategies: directly disrupting FGF1 binding to FGFR, and weakening its interaction with the essential cofactor, heparin. Such a weakening of the receptor complex may lead to incomplete conformational changes, formation of asymmetric dimers, altered recruitment of kinase adaptor proteins, or changes in the kinetics of downstream signaling pathways [7, 44, 45]. FGFRs are known to interact with a diverse array of co-receptors that modulate ligand binding, receptor activation, and signaling specificity [23]. Beyond heparan sulfate proteoglycans, which stabilize FGFR complexes and enable endocrine FGF signaling, FGFRs can also interact with Klotho proteins, galectins, N-glycans, and adhesion molecules, all of which influence receptor clustering and downstream signaling [1, 3, 46–48]. Consequently, the existence of additional, yet-to-be-characterized cofactors that influence FGFR regulation cannot be excluded.

Despite the highly promising properties demonstrated by our developed mutants, it is important to acknowledge certain limitations of this study. The precise molecular mechanisms underlying FGF1-induced glucose uptake, particularly the intricate distinction between metabolic and mitogenic signaling pathways, remain partially elusive [6, 20]. Most of our findings were derived from in vitro assays and diabetic animal models, which may not fully recapitulate the complexities of human metabolic regulation or FGF1:FGFR interactions. Although the selected FGF1 variants exhibited significantly reduced proliferative activity and improved pharmacokinetic profiles, their long-term stability, immunogenicity, and safety require precise assessment in preclinical and clinical studies. Moreover, excessively stabilizing growth factors can potentially interfere with their intrinsic regulatory mechanisms, leading to aberrant cellular responses. For instance, in the case of FGF10, an inherently unstable morphogen essential for organogenesis, engineered stabilization resulted in ectopic signaling [34]. Therefore, while both variants demonstrated antidiabetic efficacy in vivo, further optimization of formulation, dosage, and delivery routes is required before clinical translation. Future studies should focus on elucidating detailed signaling mechanisms and performing extended preclinical safety assessments to validate the therapeutic potential of these FGF1 muteins.

In summary, in this study we successfully engineered FGF1 variants that effectively uncouple potent glucose-lowering effects from undesired mitogenic activity, addressing a critical hurdle for its therapeutic application in diabetes. Our lead variants, Q40P/S47I/H93G/L135D and Q40P/S47I/H93G/S99A/K118E, demonstrate significantly reduced proliferative potential, preserved strong antidiabetic efficacy in vivo, enhanced thermodynamic stability, and superior pharmacokinetic profiles, compared to wild-type FGF1. These findings not only validate a novel strategy for designing safer FGF1-based therapeutics but also contribute to our understanding of differential FGF1 signaling. While further preclinical and clinical assessments are warranted to fully elucidate long-term safety and efficacy, these engineered FGF1 proteins represent highly promising candidates for a new generation of protein-based drugs to combat type 2 diabetes.

## Materials and methods

### Antibodies, purification columns and other reagents

The following primary antibodies were used: anti-phospho-FGFR (Tyr653/Tyr654) (pFGFR) (#06–1433) from Millipore, anti-tubulin (#T6557) from Sigma-Aldrich, anti-FGFR1 (FGFR1) (#9740), anti-phospho-p44/42 (Thr202/Tyr204) MAP kinase (pERK1/2) (#9101), anti-p44/42 MAP kinase (ERK1/2) (#9102), anti-phospho-FRS2α (Tyr196) (pFRS2) (#3864), anti-vinculin (#13901), anti-phospho-PLCγ1 (Tyr783) (pPLCγ) (#14,008), anti-PLCγ1 (#5690), anti-Glut1 (#73,015) and anti-FGF1 (#3139) from Cell Signaling Technology. Horseradish peroxidase-conjugated secondary antibodies were obtained from Jackson ImmunoResearch. The chemiluminescent substrate was sourced from Thermo Fisher Scientific. HiTrap Heparin HP and HisTrap FF columns were from GE Healthcare. Human FGF1 ELISA Kit was purchased from R&D Systems (#DFA00B).

### Cell lines

Mouse embryo fibroblast cells (NIH 3T3, CRL-1658), obtained from American Type Culture Collection (ATCC), were cultured in Dulbecco’s modified Eagle’s medium (DMEM, Thermo Fisher Scientific) supplemented with 10% bovine serum (BS, Thermo Fisher Scientific) and antibiotics (100 U/mL penicillin, 100 μg/mL streptomycin, Biowest). 3T3-L1 cells (CL-173), also from ATCC, were cultured in DMEM with reduced sodium bicarbonate (NaHCO_3_, 1.5 g/L, PAN-Biotech GmbH) containing 10% bovine serum (BS) and antibiotics (100 U/mL penicillin, 100 μg/mL streptomycin). Cell differentiation was induced when cells reached 90% confluence by replacing the medium with DMEM supplemented with 10% fetal bovine serum (FBS, Thermo Fisher Scientific), 0.5 mM isobutylmethylxanthine (Sigma-Aldrich), 1 μg/mL insulin (Sigma-Aldrich) and 1 μM dexamethasone (Sigma-Aldrich) for 3 days. Cultures were subsequently maintained in DMEM containing 10% FBS and 1 μg/mL insulin for differentiation until days 10–12. Immortalized mouse myoblast cells (C2C12, CRL-1772) and human breast adenocarcinoma cells (MCF7, HTB-22), also from ATCC, were cultured in Dulbecco’s modified Eagle’s medium (DMEM, Biowest) supplemented with 10% FBS and antibiotics (100 U/mL penicillin, 100 μg/mL streptomycin). Rhesus monkey epithelial bronchial cells (4MBr-5, CCL-208) from ATCC were cultured in Ham’s F-12 K (Kaighn’s) medium (Thermo Fisher Scientific) supplemented with antibiotics (100 U/ml penicillin, 100 μg/ml streptomycin) and 30 ng/mL EGF (Sigma-Aldrich). Western blot analysis confirmed that the cell lines used in the study do not show endogenous expression of FGF1 (Fig. S9).

### Design of FGF1 variants

Two strategies were employed to disrupt the interactions of FGF1 with FGFR at the cell surface: (i) mutations of amino acid residues involved in direct binding to the receptor and (ii) substitution of a residue involved in the interaction of FGF1 with heparin, which enhances the stability of the FGF:FGFR complex. Interactions between FGFs, FGFRs and heparin were analyzed with LigPlot software [49] using spatial structures available in PDB (Protein Data Bank) (FGF1:FGFR2:heparin [1E0O], FGF1:FGFR1 [1EVT]; FGF2:FGFR1 [1CVS]; FGF2:FGFR1:heparin [1FQ9]; FGF1:FGFR1:heparin [3OJV]. This enabled precise identification of the amino acid residues forming electrostatic, hydrogen and van der Waals bonds between individual molecules. In addition, the location of secondary structures in FGF family proteins was analyzed using ProCheck software [50], taking into account the preference of amino acid residues for specific secondary structures. Furthermore, the percentage of amino acid occurrence at selected positions was calculated based on the alignment of 845 FGF1 homologous sequences obtained from the Pfam database [51].

### Constructs, expression and purification of recombinant proteins

Previously described plasmids pET3c‐FGF1 (Ala-FGF1^22−155^) or pET3c‐FGF1-6 × His [24] served as templates for site‐directed mutagenesis. Mutations were introduced using primers (Genomed) listed in Table S1 and Phusion Hot Start II High-Fidelity PCR Master Mix (Thermo Fisher Scientific) in a QuikChange site-directed mutagenesis reaction under the conditions presented in Table S2 and Table S3. Constructs for variants: Y15A, Y94A, N95A, Y94A/N95A, K118E, L133A, R35E, R35E/Q40P/S47I/H93G and Q40P/S47I/H93G were obtained from the Department of Protein Engineering repository [24, 25, 27, 30]. Additionally, three multiple variant constructs (Q40P/S47I/H93G/L135D, Q40P/S47I/H93G/S99A and Q40P/S47I/H93G/S99A/K118E) were synthesized by GeneUniversal. Recombinant FGF1 muteins were expressed in *E. coli* BL21(DE3)pLysS (Agilent Technologies) and purified as previously described [25, 26]. The Fc-fused extracellular domain of FGFR1 (IIIc) was expressed in a CHO expression system and purified using Protein A resin as previously described [52].

### Analysis of the proliferative properties of FGF1 variants

To verify the proliferative potential of FGF1 variants, serum-starved NIH 3T3, C2C12, MCF7 and 4MBr-5 (0.5% FBS, no EGF) cells cultured in 96-well plates. Cells were treated with increasing concentrations (0.1, 1, 10 and 100 ng/mL) of FGF1 proteins in the presence of heparin (10 U/mL, Sigma Aldrich) and incubated for 48 h (NIH 3T3, C2C12), 72 h (MCF7) or 96 h (4MBr-5). AlamarBlue Cell Viability Reagent or PrestoBlue Cell Viability Reagent (Thermo Fisher Scientific) was added 4 h or 1 h, respectively, before the end of incubation. Cell viability was assessed by measuring fluorescence emission at 590 nm upon excitation at 560 nm using an Infinite M1000 PRO plate reader (Tecan). The EC_50_ parameter (half maximal effective concentration) was calculated using GraFit software (Erithacus Software). Alternatively, under the same conditions as described above, the viability of C2C12, MCF7, and 4MBr-5 cells was analyzed using the CellTiter-Fluor assay (Promega) according to the manufacturer’s protocol. Cell viability was assessed by measuring fluorescence emission at 505 nm upon excitation at 400 nm using an Infinite M1000 PRO plate reader. FGF1 mutant proliferative activity was also assessed by cell counting. For this purpose, C2C12, MCF7 and 4MBr-5 cells were seeded on a 96-well black, optically clear flat-bottom PhenoPlate (Revvity). After incubation (48 h for C2C12 cells, 72 h for MCF7 cells, and 96 h for 4MBr-5 cells), cells were washed with PBS and fixed with 4% PFA for 15 min at room temperature. Cells were then washed three times with PBS, and NucBlue dye (Thermo Fisher Scientific) was added according to the manufacturer’s instructions. The fixed and stained cells were analyzed with quantitative microscopy using the Opera Phoenix Plus High-Content Screening System (Revvity). Image acquisition from two fields of view per well was performed in normal mode with a 10 × air objective (NA 0.3), binning 2, and two peak autofocus. Harmony High-Content Imaging and Analysis Software (version 5.1; Revvity) was used for image acquisition and analysis. The number of cells was determined by counting NucBlue-positive nuclei.

### Cell signaling analysis

To analyze the ability of individual FGF1 mutants to activate signaling pathways, serum-starved NIH 3T3 cells were stimulated with 10 ng/mL of individual proteins for 15 min in the presence of 10 U/mL heparin. Cells were then lysed with SDS sample buffer and sonicated. Total cell lysates were subjected to SDS-PAGE and western blotting analysis. Fiji software [53] was used to quantify the intensity of the bands of interest. The intensity of the bands corresponding to phospho-proteins was normalized to the intensity of the total protein bands and then expressed as a fraction of the response observed for wild-type FGF1.

### Glucose uptake assay

Differentiated 3T3-L1 cells seeded on the BioCoat™ poly-D-Lysine 96-well plates (Corning) were stimulated with 20 ng/mL of FGF1 variants in the presence of heparin (10 U/mL) in DMEM without glucose (Thermo Fisher Scientific) for 20 h. Glucose uptake was determined using the bioluminescent Glucose Uptake-Glo™ Assay (Promega) according to the manufacturer’s protocol. Results were normalized to the values obtained for FGF1 WT.

### Glut1 expression analysis

Differentiated 3T3-L1 cells were treated with 100 ng/mL of selected FGF1 variants in the presence of 10 U/mL of heparin and incubated at 37 °C for 24 h in serum-free media. Cells were then lysed with SDS sample buffer and subjected to SDS-PAGE and western blotting analysis with anti-Glut1 antibody. γ-Tubulin was used as a reference protein. Fiji software was used to quantify the intensity of the bands of interest. Densitometric results were normalized to FGF1 WT.

### Proteolytic stability of FGF1 proteins in cell culture

Differentiated 3T3-L1 cells were treated with selected variants of FGF1 (1000 ng/mL) and incubated at 37 °C. At time points 0, 24, 48, 72, 96 and 120 h, media were collected and analyzed by western blotting with anti-FGF1 antibody. Densitometric analysis was performed using Fiji software. The upper band was quantified relative to the total protein at a given time point.

### Thermal stability measurements

Circular dichroism (CD) and fluorescence spectroscopy (FL) were used to determine the thermal stability of FGF1 variants. Measurements were carried out at protein concentration of 3–4 μM in the presence of 0.7 M GdmCl in 25 mM sodium phosphate buffer, pH 7.3. Thermal denaturation curves were obtained from changes in the ellipticity signal at 228 nm on a Jasco J-715 spectropolarimeter and the emission signal at 353 nm of a single tryptophan residue excited at 280 nm on a Jasco FP-750 spectrofluorometer [26]. Thermodynamic data were analyzed using PeakFit software (Jandel Scientific Software), assuming a two-state reversible equilibrium transition.

### Analysis of the interaction between FGF1 variants and FGFR1

To analyze the binding affinity of FGF1 variants to FGFR1 (IIIc), a biolayer interferometry (BLI) technique was applied using a ForteBio Octet K2 instrument (Pall ForteBio), as previously described [30]. All steps of the interaction studies were performed at 25 °C with shaking at 1000 rpm in PBS. Protein A biosensors were loaded with recombinant extracellular domain of FGFR1 (IIIc) fused to Fc fragment (20 μg/mL for 600 s). The association and dissociation phases (180 s each) were monitored at different concentrations of FGF1 variants ranging from 100 to 800 nM. Equilibrium dissociation constants (KD) were calculated from fitted steady-state curves with ForteBio Data Analysis 11.0 software (Pall ForteBio).

### In vivo study

All animal experiments were conducted in accordance with the guidelines of the Local Ethics Committees for Animal Experimentation (Bydgoszcz/13/2018; Wroclaw/04/2017). Mice aged 9–10 weeks were randomly allocated to the experimental groups to minimize selection bias, with 6–7 individuals in each group, and housed in a temperature-controlled environment with a 12-h light/12-h dark cycle. Male *db/db* mice (BKS.Cg- + Lepr^db^/+ Lepr^db^/OlaHsd) from Charles Rivers or Envigo were fed Teklad Global Diet 2018 (Envigo) or 5K52 (Charles Rivers) and acidified water ad libitum. Blood glucose levels were monitored using a standard Accu-Chek®Performa glucometer (Roche), and samples were collected by tail bleeding. An initial non-fasting hyperglycemic condition was confirmed at blood glucose level > 250 mg/dL. FGF1 variants were injected subcutaneously at a dose of 1 mg/kg, with phosphate-buffered saline injected as a control, and blood glucose levels were monitored as a function of time. The body weight of the animals was measured 96 h after FGF1 administration. A single-dose pharmacokinetic study was conducted in 8- to 10-weeks-old Wistar Han rats obtained from the in-house breeding colony at TAZD-CBU (Medical University of Gdansk). The animals were housed under continuously controlled conditions, with a 12-h light/12-h dark cycle at 22 °C, with constant access to feed and water ad libitum. The experiment was conducted after the animals were placed under general anesthesia (inhalation with a mixture of isoflurane and oxygen). FGF1 variants were administered subcutaneously to rats (5 animals per group) at a dose of 0.5 mg/kg. Blood samples were collected at the following time points: 0, 5, 15, and 30 min, as well as 1, 2, 4, 7, 12, 24, and 48 h post-injection. Serum FGF1 concentrations were quantified using ELISA according to the manufacturer’s protocol.

### Statistical analysis

All experiments were performed in at least triplicate. Values of n express the actual number of independent experiments. An unpaired, two-tailed Student's t-test was used to statistically evaluate differences. Results were considered significant for values of *p* ≤ 0.05.

## Supplementary Information

Supplementary Material 1.

## Data Availability

All data generated or analyzed in the study are available from the corresponding authors on reasonable request.

## Abbreviations

AUC: Area under the curve
BLI: Biolayer interferometry
C_max_: Maximum concentration
EC_50_: Half maximal effective concentration
ΔH_den_: Denaturation enthalpy change
ERK 1/2: extracellular signal-regulated protein kinases 1 and 2
FGF1: Fibroblast growth factor 1
FGFR: Fibroblast growth factor receptor
FRS2: Fibroblast growth factor receptor substrate 2
GdmCl: Guanidinium chloride
Glut1: Glucose transporter 1
HSPGs: Heparan sulfate proteoglycans
K_D_: Equilibrium dissociation constants
PDB: Protein Data Bank
PKCδ: Protein kinase C delta
PLCγ: Phospholipase C gamma
T2D: Type 2 diabetes
T_den_: Denaturation temperature
T_max_: Time of maximum drug concentration

## References

[1] Beenken A, Mohammadi M. The FGF family: biology, pathophysiology and therapy. Nat Rev Drug Discov. 2009;8:235–53. 10.1038/nrd2792. 19247306 10.1038/nrd2792 PMC3684054

[2] Nies VJ, Sancar G, Liu W, van Zutphen T, Struik D, Yu RT, et al. Fibroblast growth factor signaling in metabolic regulation. Front Endocrinol. 2016;6:193. 10.3389/fendo.2015.00193. 10.3389/fendo.2015.00193 PMC4718082 26834701

[3] Ornitz DM, Itoh N. The fibroblast growth factor signaling pathway. WIREs Dev Biol. 2015;4:215–66. 10.1002/wdev.176. 10.1002/wdev.176 PMC4393358 25772309

[4] Goetz R, Mohammadi M. Exploring mechanisms of FGF signalling through the lens of structural biology. Nat Rev Mol Cell Biol. 2013;14:166–80. 10.1038/nrm3528. 23403721 10.1038/nrm3528 PMC3695728

[5] Zhang X, Ibrahimi OA, Olsen SK, Umemori H, Mohammadi M, Ornitz DM. Receptor specificity of the fibroblast growth factor family: The complete mammalian FGF family. J Biol Chem. 2006;281:15694–700. 10.1074/jbc.M601252200. 16597617 10.1074/jbc.M601252200 PMC2080618

[6] Huang Z, Tan Y, Gu J, Liu Y, Song L, Niu J, et al. Uncoupling the mitogenic and metabolic functions of FGF1 by tuning FGF1-FGF receptor dimer stability. Cell Rep. 2017;20:1717–28. 10.1016/j.celrep.2017.06.063. 28813681 10.1016/j.celrep.2017.06.063 PMC5821125

[7] Mohammadi M, Zinkle A. A threshold model for receptor tyrosine kinase signaling specificity and cell fate determination. F1000Research. 2018;7:1–16. 10.12688/f1000research.14143.1 10.12688/f1000research.14143.1 PMC6013765 29983915

[8] Itoh N, Ohta H, Konishi M. Endocrine FGFs: evolution, physiology, pathophysiology, and pharmacotherapy. Front Endocrinol. 2015;6:1–9. 10.3389/fendo.2015.00154. 10.3389/fendo.2015.00154 PMC4586497 26483756

[9] Scarlett JM, Rojas JM, Matsen ME, Kaiyala KJ, Stefanovski D, Bergman RN, et al. Central injection of fibroblast growth factor 1 induces sustained remission of diabetic hyperglycemia in rodents. Nat Med. 2016;22:800–6. 10.1038/nm.4101. 27213816 10.1038/nm.4101 PMC4938755

[10] Suh JM, Jonker JW, Ahmadian M, Goetz R, Lackey D, Osborn O, et al. Endocrinization of FGF1 produces a neomorphic and potent insulin sensitizer. Nature. 2014;513:436–9. 10.1038/nature13540. 25043058 10.1038/nature13540 PMC4184286

[11] Jin L, Yang R, Geng L, Xu A. Fibroblast growth factor-based pharmacotherapies for the treatment of obesity-related metabolic complications. Annu Rev Pharmacol Toxicol. 2023;63:359–82. 10.1146/annurev-pharmtox-032322-093904. 36100222 10.1146/annurev-pharmtox-032322-093904

[12] Jonker JW, Suh JM, Atkins AR, Ahmadian M, Li P, Whyte J, et al. A PPARγ-FGF1 axis is required for adaptive adipose remodelling and metabolic homeostasis. Nature. 2012;485:391–4. 10.1038/nature10998. 22522926 10.1038/nature10998 PMC3358516

[13] Perry RJ, Lee S, Ma L, Zhang D, Schlessinger J, Shulman GI. FGF1 and FGF19 reverse diabetes by suppression of the hypothalamic-pituitary-adrenal axis. Nat Commun. 2015;6:1–10. 10.1038/ncomms7980. 10.1038/ncomms7980 PMC4413509 25916467

[14] Scarlett JM, Muta K, Brown JM, Rojas JM, Matsen ME, Acharya NK, et al. Peripheral mechanisms mediating the sustained antidiabetic action of FGF1 in the brain. Diabetes. 2019;68:654–64. 10.2337/db18-0498. 30523024 10.2337/db18-0498 PMC6385755

[15] Tennant KG, Lindsley SR, Kirigiti MA, True C, Kievit P. Central and peripheral administration of fibroblast growth factor 1 improves pancreatic islet insulin secretion in diabetic mouse models. Diabetes. 2019;68:1462–72. 10.2337/db18-1175. 31048370 10.2337/db18-1175 PMC6609981

[16] Liang G, Song L, Chen Z, Qian Y, Xie J, Zhao L, et al. Fibroblast growth factor 1 ameliorates diabetic nephropathy by an anti-inflammatory mechanism. Kidney Int. 2018;93:95–109. 10.1016/j.kint.2017.05.013. 28750927 10.1016/j.kint.2017.05.013 PMC5818994

[17] Wang A, Yan X, Zhang C, Du C, Long W, Zhan D, et al. Characterization of fibroblast growth factor 1 in obese children and adolescents. Endocr Connect. 2018;7:932–40. 10.1530/EC-18-0141. 30299902 10.1530/EC-18-0141 PMC6130312

[18] Wang S, Yang Q, Yu S, Pan R, Jiang D, Liu Y, et al. Fibroblast growth factor 1 levels are elevated in newly diagnosed type 2 diabetes compared to normal glucose tolerance controls. Endocr J. 2016;63:359–65. 10.1507/endocrj.EJ15-0627. 26806193 10.1507/endocrj.EJ15-0627

[19] Zhu J, Wang Y, Zhu K, Gao J, Wan X, Pang X, et al. Serum fibroblast growth factor 1 is associated with the decreased risk of obesity in human. Exp Clin Endocrinol Diabetes. 2017;125:322–6. 10.1055/s-0043-104532. 28303556 10.1055/s-0043-104532

[20] Nies VJM, Struik D, Liu S, Liu W, Kruit JK, Downes M, et al. Autocrine FGF1 signaling promotes glucose uptake in adipocytes. Proc Natl Acad Sci USA. 2022;119(40):e2122382119. 10.1073/pnas.2122382119. 36161959 10.1073/pnas.2122382119 PMC9546606

[21] Presta M, Chiodelli P, Giacomini A, Rusnati M, Ronca R. Fibroblast growth factors (FGFs) in cancer: FGF traps as a new therapeutic approach. Pharmacol Ther. 2017;179:171–87. 10.1016/j.pharmthera.2017.05.013. 28564583 10.1016/j.pharmthera.2017.05.013

[22] Xia X, Kumru OS, Blaber SI, Middaugh CR, Li L, Ornitz DM, et al. An S116R phosphorylation site mutation in human fibroblast growth factor-1 differentially affects mitogenic and glucose-lowering activities. J Pharm Sci. 2016;105:3507–19. 10.1016/j.xphs.2016.09.005. 27773526 10.1016/j.xphs.2016.09.005 PMC5310217

[23] Chen G, Chen L, Li X, Mohammadi M. FGF-based drug discovery: advances and challenges. Nat Rev Drug Discov. 2025;24:335–57. 10.1038/s41573-024-01125-w. 39875570 10.1038/s41573-024-01125-w

[24] Zakrzewska M, Wiedlocha A, Szlachcic A, Krowarsch D, Otlewski J, Olsnes S. Increased protein stability of FGF1 can compensate for its reduced affinity for heparin. J Biol Chem. 2009;284:25388–403. 10.1074/jbc.M109.001289. 19574212 10.1074/jbc.M109.001289 PMC2757240

[25] Zakrzewska M, Krowarsch D, Wiedlocha A, Otlewski J. Design of fully active FGF-1 variants with increased stability. Protein Eng Des Sel. 2004;17:603–11. 10.1093/protein/gzh076. 15469994 10.1093/protein/gzh076

[26] Zakrzewska M, Krowarsch D, Wiedlocha A, Olsnes S, Otlewski J. Highly stable mutants of human fibroblast growth factor-1 exhibit prolonged biological action. J Mol Biol. 2005;352:860–75. 10.1016/j.jmb.2005.07.066. 16126225 10.1016/j.jmb.2005.07.066

[27] Zakrzewska M, Krowarsch D, Wiedlocha A, Olsnes S, Otlewski J. Structural requirements of FGF-1 for receptor binding and translocation into cells. Biochemistry. 2006;45:15338–48. 10.1021/bi0618114. 17176056 10.1021/bi0618114

[28] Bailly K, Soulet F, Leroy D, Amalric F, Bouche G. Uncoupling of cell proliferation and differentiation activities of basic fibroblast growth factor. FASEB J. 2000;14:333–44. 10.1096/fasebj.14.2.333. 10657989

[29] Mori S, Wu CY, Yamaji S, Saegusa J, Shi B, Ma Z, et al. Direct binding of integrin αvβ3 to FGF1 plays a role in FGF1 signaling. J Biol Chem. 2008;283:18066–75. 10.1074/jbc.M801213200. 18441324 10.1074/jbc.M801213200 PMC2440593

[30] Szlachcic A, Sochacka M, Czyrek A, Opalinski L, Krowarsch D, Otlewski J, et al. Low stability of integrin-binding deficient mutant of FGF1 restricts its biological activity. Cells. 2019;8:899. 10.3390/cells8080899. 31443196 10.3390/cells8080899 PMC6721657

[31] Wiedłocha A, Nilsen T, Wesche J, Sørensen V, Małecki J, Marcinkowska E, et al. Phosphorylation-regulated nucleocytoplasmic trafficking of internalized fibroblast growth factor-1. Mol Biol Cell. 2005;16:794–810. 10.1091/mbc.e04-05-0389. 15574884 10.1091/mbc.E04-05-0389 PMC545912

[32] Huang J, Wang K, Shiflett LA, Brotto L, Bonewald LF, Wacker MJ, et al. Fibroblast growth factor 9 (FGF9) inhibits myogenic differentiation of C2C12 and human muscle cells. Cell Cycle. 2019;18:3562–80. 10.1080/15384101.2019.1691796. 31735119 10.1080/15384101.2019.1691796 PMC6927711

[33] Nilsson EM, Brokken LJS, Härkönen PL. Fibroblast growth factor 8 increases breast cancer cell growth by promoting cell cycle progression and by protecting against cell death. Exp Cell Res. 2010;316:800–12. 10.1016/j.yexcr.2009.11.019. 19962979 10.1016/j.yexcr.2009.11.019

[34] Czyrek AA, Baran K, Hruba E, Horackova A, Bosakova V, Chudzian J, et al. Increased thermal stability of FGF10 leads to ectopic signaling during development. Cell Mol Life Sci. 2025;82:167. 10.1007/s00018-025-05681-1. 40257501 10.1007/s00018-025-05681-1 PMC12011707

[35] Casar JC, Cabello-Verrugio C, Olguin H, Aldunate R, Inestrosa NC, Brandan E. Heparan sulfate proteoglycans are increased during skeletal muscle regeneration: requirement of syndecan-3 for successful fiber formation. J Cell Sci. 2004;117:73–84. 10.1242/jcs.00828. 14627628 10.1242/jcs.00828

[36] DePaoli AM, Zhou M, Kaplan DD, Hunt SC, Adams TD, Learned RM, et al. FGF19 analog as a surgical factor mimetic that contributes to metabolic effects beyond glucose homeostasis. Diabetes. 2019;68:1315–28. 10.2337/db18-1305. 30862680 10.2337/db18-1305

[37] Gaich G, Chien JY, Fu H, Glass LC, Deeg MA, Holland WL, et al. The effects of LY2405319, an FGF21 analog, in obese human subjects with type 2 diabetes. Cell Metab. 2013;18:333–40. 10.1016/j.cmet.2013.08.005. 24011069 10.1016/j.cmet.2013.08.005

[38] Harrison SA, Neff G, Guy CD, Bashir MR, Paredes AH, Frias JP, et al. Efficacy and safety of Aldafermin, an engineered FGF19 analog, in a randomized, double-blind, placebo-controlled trial of patients with nonalcoholic steatohepatitis. Gastroenterology. 2021;160:219-231.e1. 10.1053/j.gastro.2020.08.004. 32781086 10.1053/j.gastro.2020.08.004

[39] Simpson RJ. Stabilization of proteins for storage. Cold Spring Harb Protoc. 2010;5. 10.1101/pdb.top79 10.1101/pdb.top79 20439424

[40] Babik S, Samsonov SA, Pisabarro MT. Computational drill down on FGF1-heparin interactions through methodological evaluation. Glycoconj J. 2017;34:427–40. 10.1007/s10719-016-9745-4. 27858202 10.1007/s10719-016-9745-4 PMC5487771

[41] Pellegrini L, Burke DF, von Delft F, Mulloy B, Blundell TL. Crystal structure of fibroblast growth factor receptor ectodomain bound to ligand and heparin. Nature. 2000;407:1029–34. 10.1038/35039551. 11069186 10.1038/35039551

[42] Zhan X, Hu X, Friesel R, Maciag T. Long term growth factor exposure and differential tyrosine phosphorylation are required for DNA synthesis in BALB/c 3T3 cells. J Biol Chem. 1993;268:9611–20. 7683656

[43] Zhao L, Niu J, Lin H, Zhao J, Liu Y, Song Z, et al. Paracrine-endocrine FGF chimeras as potent therapeutics for metabolic diseases. EBioMedicine. 2019;48:462–77. 10.1016/j.ebiom.2019.09.052. 31631034 10.1016/j.ebiom.2019.09.052 PMC6838362

[44] Karl K, Paul MD, Pasquale EB, Hristova K. Ligand bias in receptor tyrosine kinase signaling. J Biol Chem. 2020;295:18494–507. 10.1074/jbc.REV120.015190. 33122191 10.1074/jbc.REV120.015190 PMC7939482

[45] Niu J, Zhao J, Wu J, Qiao G, Gu J, Zhou C, et al. Curtailing FGF19’s mitogenicity by suppressing its receptor dimerization ability. Proc Natl Acad Sci U S A. 2020;117:29025–34. 10.1073/pnas.2010984117. 33144503 10.1073/pnas.2010984117 PMC7682408

[46] Kurosu H, Ogawa Y, Miyoshi M, Yamamoto M, Nandi A, Rosenblatt KP, et al. Regulation of fibroblast growth factor-23 signaling by Klotho. J Biol Chem. 2006;281:6120–3. 10.1074/jbc.C500457200. 16436388 10.1074/jbc.C500457200 PMC2637204

[47] Latko M, Czyrek A, Porębska N, Kucińska M, Otlewski J, Zakrzewska M, et al. Cross-talk between fibroblast growth factor receptors and other cell surface proteins. Cells. 2019;8:455. 10.3390/cells8050455. 31091809 10.3390/cells8050455 PMC6562592

[48] Zukowska D, Gedaj A, Porebska N, Pozniak M, Krzyscik M, Czyrek A, et al. Receptor clustering by a precise set of extracellular galectins initiates FGFR signaling. Cell Mol Life Sci. 2023;80(4):113. 10.1007/s00018-023-04768-x. 37012400 10.1007/s00018-023-04768-x PMC10070233

[49] Wallace AC, Laskowski RA, Thornton JM. Ligplot: a program to generate schematic diagrams of protein-ligand interactions. Protein Eng Des Sel. 1995;8:127–34. 10.1093/protein/8.2.127. 10.1093/protein/8.2.127 7630882

[50] Laskowski RA, MacArthur MW, Moss DS, Thornton JM. PROCHECK: a program to check the stereochemical quality of protein structures. J Appl Crystallogr. 1993;26:283–91. 10.1107/S0021889892009944.

[51] Bateman A, Coin L, Durbin R, Finn RD, Hollich V, Griffiths-Jones S, et al. The Pfam protein families database. Nucleic Acids Res. 2004;32. 10.1093/nar/gkh121 10.1093/nar/gkh121 PMC308855 14681378

[52] Sokolowska-Wedzina A, Borek A, Chudzian J, Jakimowicz P, Zakrzewska M, Otlewski J. Efficient production and purification of extracellular domain of human FGFR-Fc fusion proteins from Chinese hamster ovary cells. Protein Expr Purif. 2014;99:50–7. 10.1016/j.pep.2014.03.012. 24727156 10.1016/j.pep.2014.03.012

[53] Schindelin J, Arganda-Carreras I, Frise E, Kaynig V, Longair M, Pietzsch T, et al. Fiji: an open-source platform for biological-image analysis. Nat Methods. 2012;9:676–82. 10.1038/nmeth.2019. 22743772 10.1038/nmeth.2019 PMC3855844

