# Supplementary material for: Engineered fibroblast growth factor 1 variants uncouple glucose-lowering effects from mitogenic activity with therapeutic potential for type 2 diabetes
Source: Mol Biomed. 2026 Jan 11;7:4. doi: 10.1186/s43556-025-00398-w (PMC12790546; doi:10.1186/s43556-025-00398-w)
Supplement: Supplementary file 1 — Supplementary Material 1. [file 43556_2025_398_MOESM1_ESM.pdf]

Supplementary Material 1

Molecular Biomedicine

Engineered fibroblast growth factor 1 variants uncouple glucose-lowering effects from mitogenic activity with therapeutic potential for type 2 diabetes

Aleksandra A. Czyrek[1,2,3,4], Daniel Krowarsch[1], Szymon Sidor[1], Michal Janiszewski[5], Ewa Drzazga-Wilk[5], Katarzyna Bazydlo-Guzenda[5], Pawel Buda[5], Jerzy Pieczykolan[5], Natalia Porebska[6], Marta Minkiewicz[1,6], Pavel Krejci[2,3,4], Maciej Wieczorek[5], Jacek Otlewski[1], Malgorzata Zakrzewska[1*]

[1]Department of Protein Engineering, Faculty of Biotechnology, University of Wroclaw, Joliot-Curie 14a, 50-383 Wroclaw, Poland

[2]Faculty of Medicine, Department of Biology, Masaryk University, Kamenice 753/5, 62500 Brno, Czech Republic

[3]International Clinical Research Center, St. Anne's University Hospital, Pekarska 53, 65691 Brno, Czech Republic

[4]Institute of Animal Physiology and Genetics of the CAS, Rumburska 89, 27721 Libechov, Czech Republic

[5]Celon Pharma S.A., R&D Centre, Marymoncka 15, 05-152, Kazun Nowy, Poland

[6]Department of Medical Biotechnology, Faculty of Biotechnology, University of Wroclaw, Joliot-Curie 14a, 50-383 Wroclaw, Poland

*Correspondence to malgorzata.zakrzewska@uwr.edu.pl

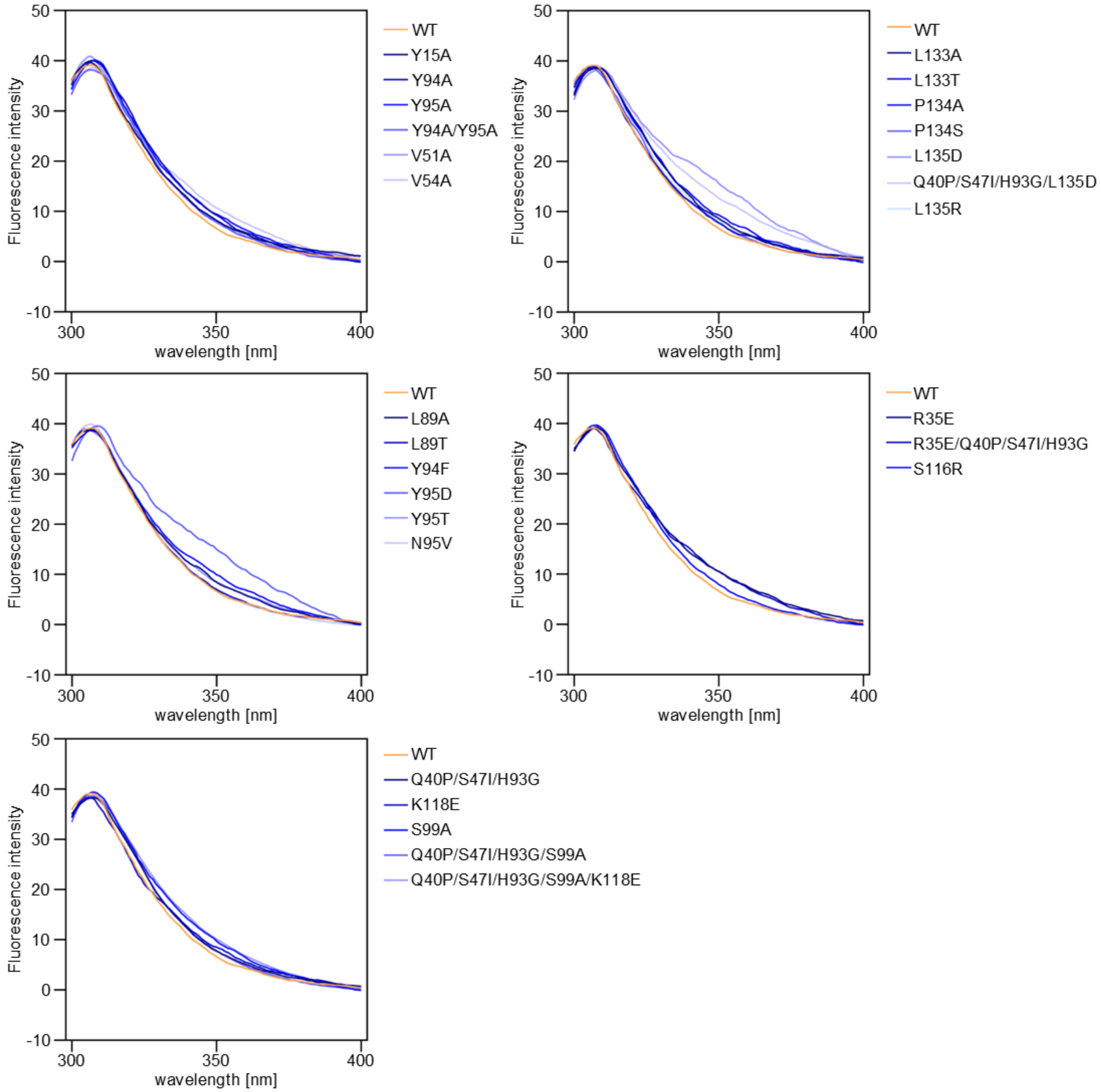

Figure S1. Fluorescence emission spectra of all purified FGF1 variants. The spectra of proteins at a concentration of 2 μM were recorded in the range of 300-400 nm after excitation at 280 nm.

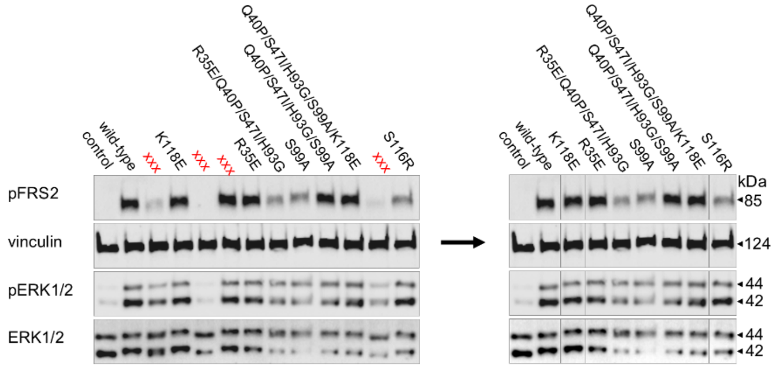

Figure S2. Original membrane subjected to WB analysis together with the version shown in Figure 2. Serum-starved NIH 3T3 cells were treated with 10 ng/mL FGF1 variants for 15 min in the presence of heparin (10 ng/mL), and activation of the downstream signaling was detected by immunoblotting using the following antibodies: anti-phospho-FRS2 (pFRS2), anti-phospho-ERK1/2 (pERK1/2). Anti-ERK1/2 and anti-vinculin antibodies provided equal loading. The lanes marked XXX concerned the analysis of protein variants not described in the publication and were cut out.

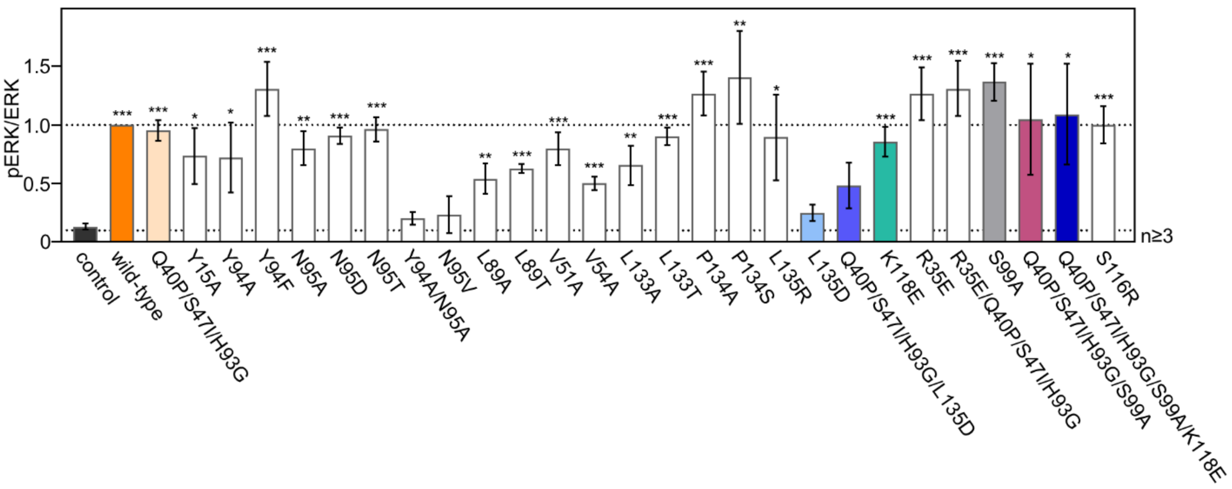

Figure S3. Densitometric analysis of western blotting result from Figue 2a. Data shown as mean ± SEM, n≥3. Serum-starved NIH 3T3 cells were treated with 10 ng/mL FGF1 variants for 15 min in the presence of heparin (10 U/mL). Statistical significance: *p ≤ 0.05; **p ≤ 0.01 and ***p ≤ 0.001.

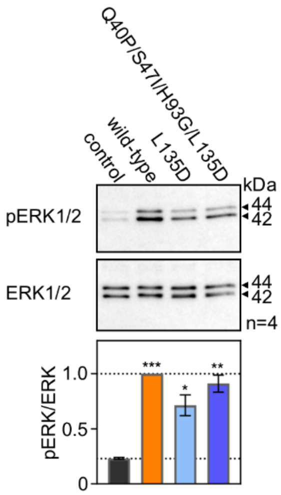

Figure S4. Western-blotting analysis of ERK activation upon stimulation of cells with elevated concentrations of FGF1 variants. Serum-starved NIH 3T3 cells were treated with 50 ng/mL of FGF1 variants for 15 min in the presence of heparin (10 U/mL), and activation of the downstream cascade was detected by immunoblotting using anti-phospho-ERK1/2 (pERK1/2). Anti-ERK1/2 antibody was used to ensure equal loading. Data shown as mean ± SEM, n=4. Statistical significance: *$p \leq 0.05$; **$p \leq 0.01$ and ***$p \leq 0.001$.

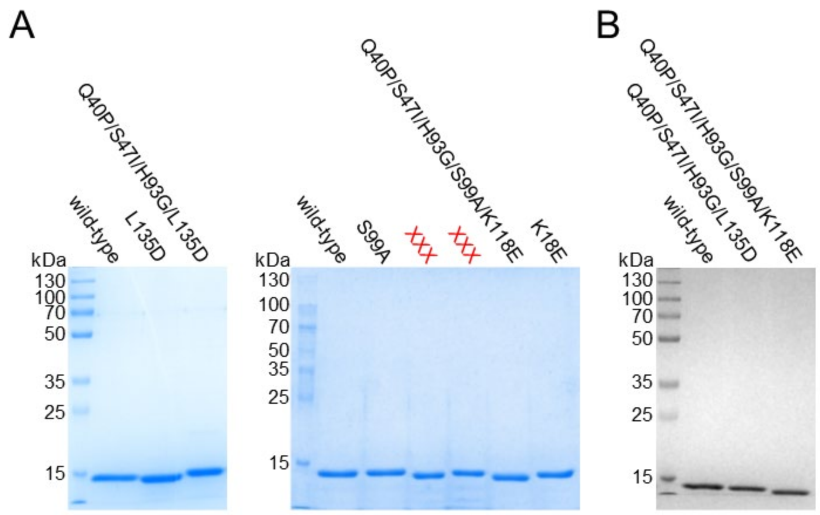

Figure S5. SDS-PAGE analysis of proteins used *in vivo* studies. Protein variants used (**A**) to assess change in blood glucose levels in *db/db* mice and (**B**) for pharmacokinetics analysis in Wistar Han rats. The purity of the proteins was assessed as > 95%. XXX - analysis of protein variants not described in the publication.

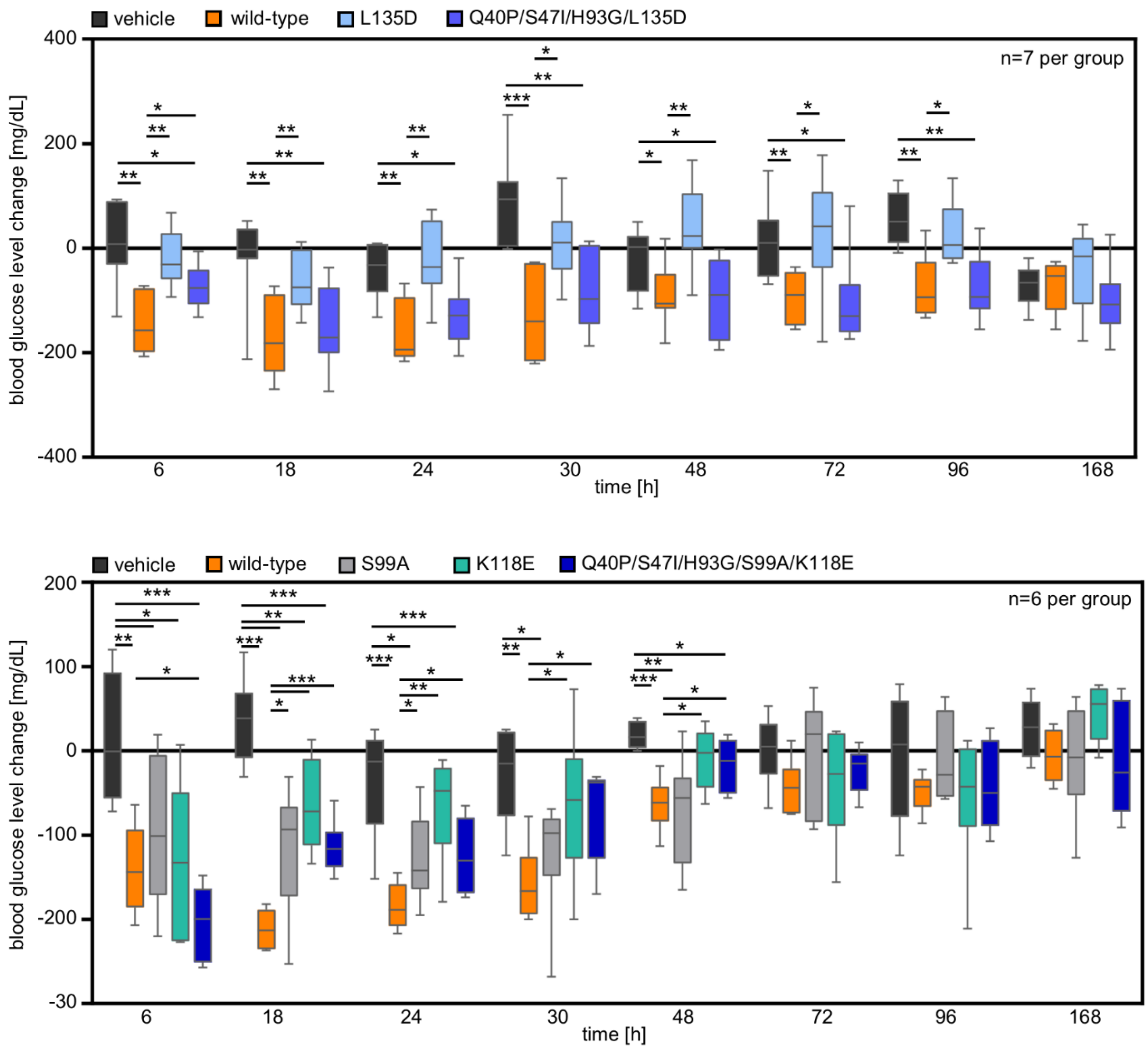

Figure S6. Box-and-whisker plot of metabolic activity of FGF1 variants *in vivo* (data from Figure 6a-b). Change in blood glucose levels in db/db mice after a single administration of FGF1 variants (measurements up to 168 h). FGF1 variants were administered at a dose of 1 mg/kg body weight. Data were normalized to glucose levels prior to protein administration and presented as mean ± SEM, n=7/6. Statistical significance $^*p \leq 0.05$; $^{**}p \leq 0.01$ and $^{***}p \leq 0.001$.

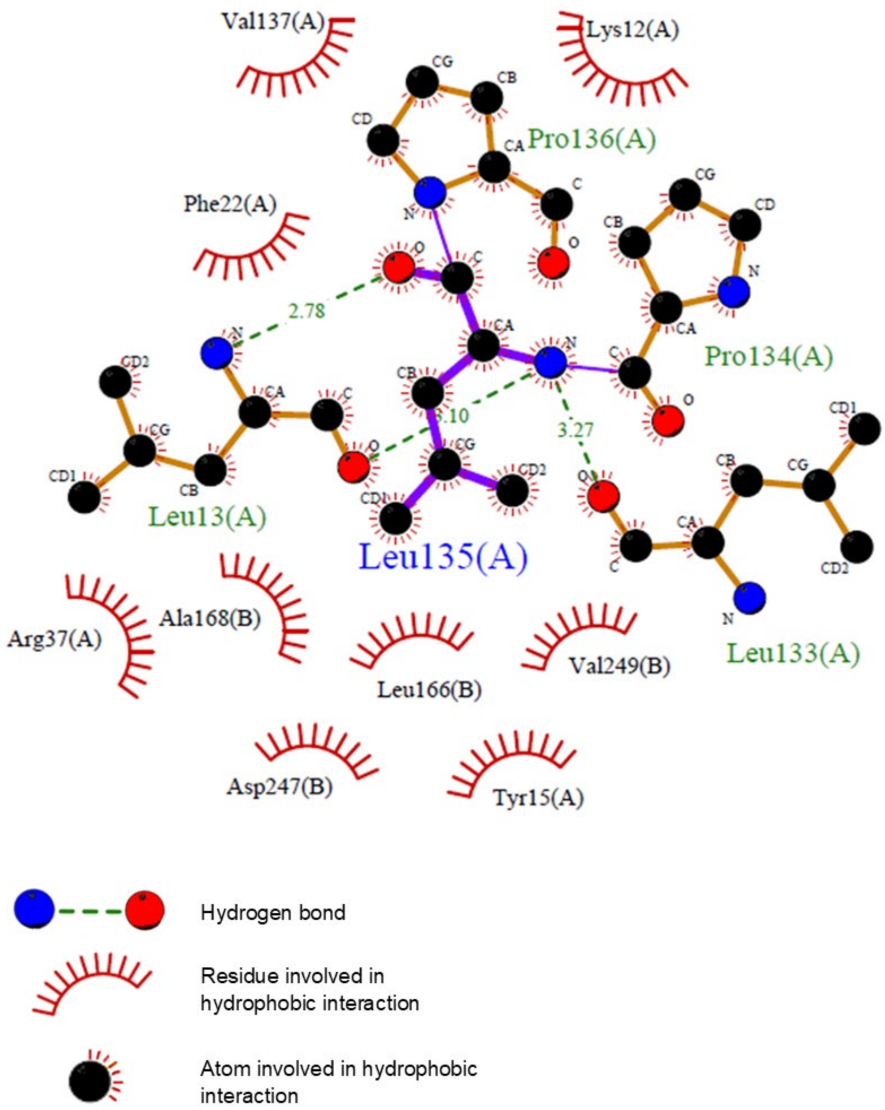

Figure S7. Schematic representation of the interaction between the Leu135 residue of FGF1 and the FGF receptor 1. The diagram was generated using Ligplot software based on the structure of the FGF1:FGFR1:heparin complex (PDB: 1e0o). The green dotted line shows hydrogen bonds, and their lengths are marked. Amino acid residues derived from FGF1 are marked with the letter A, while residues from FGFR1 are marked with the letter B.

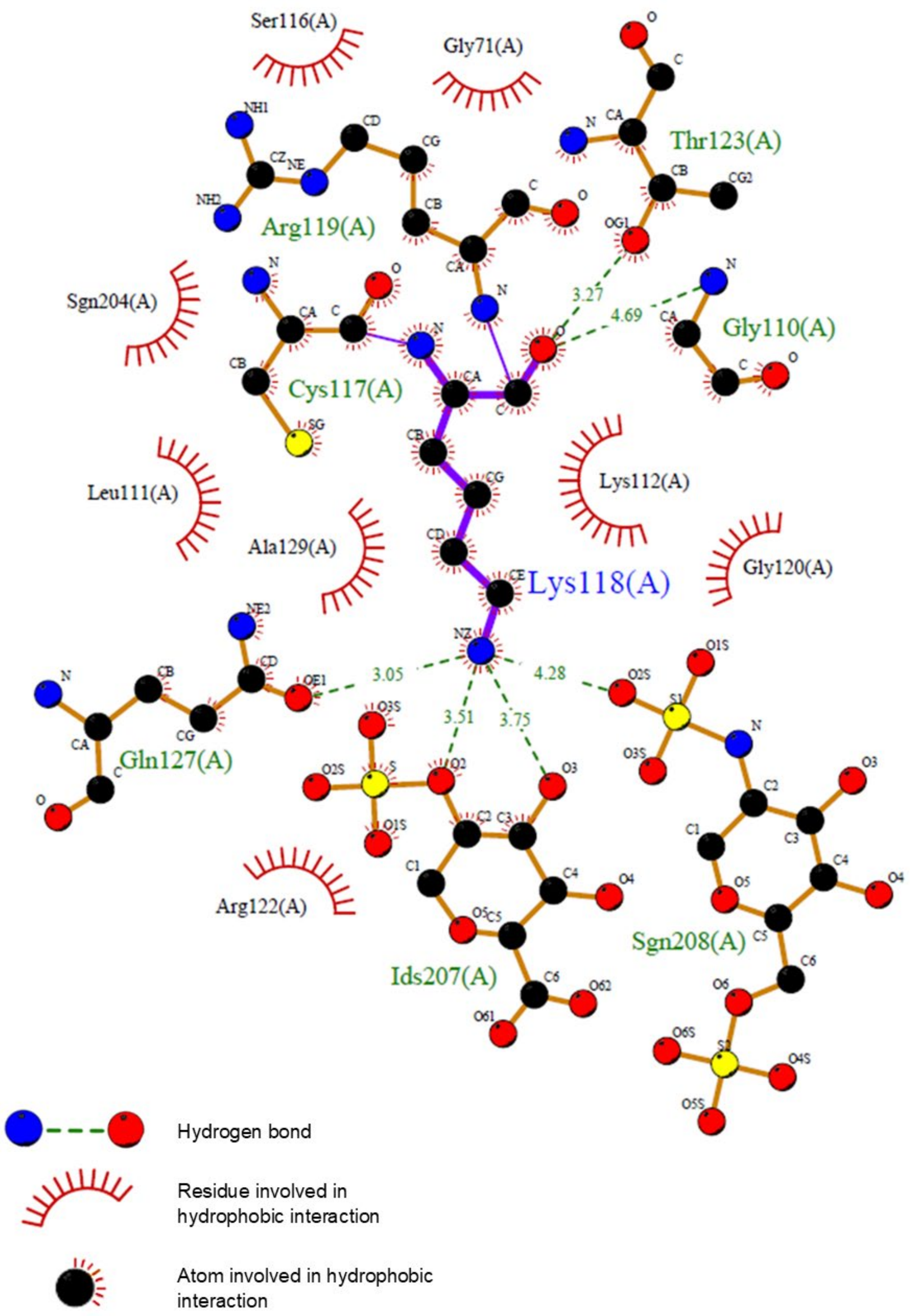

Figure S8. Schematic representation of the interaction between the Lys118 residue of FGF1 and heparin. The diagram was generated using Ligplot software based on the structure of the FGF1:FGFR1:heparin complex (PDB: 1e0o). The green dotted line shows hydrogen bonds, and their lengths are marked. Amino acid residues derived from FGF1 and the heparin fragments (Ids207 and Sgn208) are marked with the letter A.

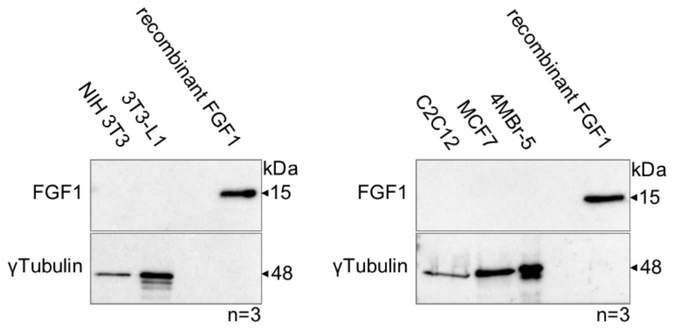

Figure S9. Analysis of FGF1 expression in 3T3-L1, NIH 3T3, C2C12, MCF7, and 4MBr-5 cells. Cell lysates were analyzed by Western blotting using specific antibodies against FGF1 and γTubulin. Recombinant FGF1 protein (5 ng) was used as a positive control, n=3.

Table S1. Thermodynamic parameters of denaturation of FGF1 variants. Denaturation was carried out in the presence of 0.7 M GdmCl. Changes in ellipticity (λ=227 nm) and fluorescence emission (λ=353 nm with λ=280 nm excitation) were monitored. T_{den} – denaturation temperature, ΔH_{den} – denaturation enthalpy change. The table shows the mean ± SEM, n=3.

	circular dichroism			fluorometry		
**FGF1 variant**	T_{den} ± SEM [°C]	ΔT_{den} [°]	ΔH_{den} [kcal mol^{-1}]	T_{den} ± SEM [°C]	ΔT_{den} [°]	ΔH_{den} [kcal mol^{-1}]
**wild-type**	40.2±0.2		65.3	39.8±0.5		66.9
**K118E**	37.5±1.1	-2.7	63	37.5±0.4	-2.3	62.3
**L135D**	20.3±0.5	-19.9	32.8	18.6±0.8	-21.2	36
**S99A**	38.0±0.2	-2.2	62.8	35.3±0.8	-4.5	63.5
**Q40P/S47I/H93G/L135D**	53.0±0.5	12.8	82.5	50.4±0.1	10.6	95.4
**Q40P/S47I/H93G/S99A**	59.6±0.2	19.4	94	57.6±0.9	17.8	101
**Q40P/S47I/H93G/S99A/K118E**	60.5±0.1	20.3	106.1	59.2±0.2	19.4	109

Table S2. FGF1 variants and their mitogenic activity in different cell types. To assess the mitogenic activity of FGF1 variants, serum-starved C2C12, MCF7 or 4MBr-5 cells were stimulated with FGF1 mutants in the concentration range of 0.1-100 ng/mL in the presence of 10 U/mL heparin for 48 h, 72h or 96 h, respectively. Data are presented as the mean of EC_{50} (ng/mL) of FGF1variants ± SEM, n=3.

**Method**	FGF1 variant	MCF7 EC_{50} ± SEM [ng/mL]	4MBr-5 EC_{50} ± SEM [ng/mL]	C2C12 EC_{50} ± SEM [ng/mL]
**PrestoBlue**				
	wild-type	0.4±0.3	0.1±0.03	3.9±0.4
	Q40P/S47I/H93G/L135D	49.7±21.3	56.3±22.7	184.3±15.1
	Q40P/S47I/H93G/S99A/K118E	13.2±3.7	52.6±8.5	7.6±1.7
**CellTiter-Fluor**				
	wild-type	1.3±0.2	1.6±0.3	2.4±0.5
	Q40P/S47I/H93G/L135D	105.5±35.7	207±93.3	92.2±22.6
	Q40P/S47I/H93G/S99A/K118E	26.9±12.2	3.5±1.7	4.6±1.7
**Cell counting**				
	wild-type	1.4±0.8	4.1±1.5	1.5±0.1
	Q40P/S47I/H93G/L135D	87.2±44.6	111.5±30.8	146.6±11.9
	Q40P/S47I/H93G/S99A/K118E	15±11.3	55.2±28.8	7.1±0.7

Table S3. Pharmacokinetic parameters of FGF1 variants in Wistar Han rats. Data are presented as mean ± SEM, n=5. Significance of the result in relation to wild-type (WT) FGF1: NS – non-significant, *p ≤ 0.05; **p ≤ 0.01 and ***p ≤ 0.001. AUC – area under the curve; C_{max} – maximum drug concentration; T_{max} – time to reach maximum drug concentration.

FGF1 variant	AUC	significancy	C_{max} [ng/mL]	significancy	T_{max} (h)	significancy
**wild-type**	277.2±21.5		59.1±10		2.4±0.4	
**Q40P/S47I/H93G/L135D**	533.1±40.3	***	141.3±15.3	**	1.6±0.2	NS
**Q40P/S47I/H93G/S99A/K118E**	1261.8±49	***	303.6±24.6	***	1.3±0.3	NS

Table S4. Sequences of primers designed to introduce substitutions in the FGF1 sequence in the site-directed mutagenesis reaction.

Substitution	Type	Sequence
**V51A**	*forward*	GCTCAGTGCGGAAAGCGCGGGGGAGGTGTATATAAAG
	reverse	CTTTATATACACCTCCCCCGCGCTTTCCGCACTGAGC
**V54A**	*forward*	GCGGAAAGCGTGGGGGAGGCGTATATAAAGAGTACCG
	reverse	CGGTACTCTTTATATACGCCTCCCCCACGCTTTCCGC
**L89A**	*forward*	GGAATGTTTGTTCCTGGAAAGGGCGGAGGAGAACCATTACAAC
	reverse	GTTGTAATGGTTCTCCTCCGCCCTTTCCAGGAACAAACATTCC
**L89T**	*forward*	GGAATGTTTGTTCCTGGAAAGGACGGAGGAGAACCATTACAAC
	reverse	GTTGTAATGGTTCTCCTCCGTCCTTTCCAGGAACAAACATTCC
**Y94F**	*forward*	GGCTGGAGGAGAACCATTTCAACACCTATATATCCAAGAAGC
	reverse	GCTTCTTGGATATATAGGTGTTGAAATGGTTCTCCTCCAGCC
**N95D**	*forward*	GGCTGGAGGAGAACCATTACGACACCTATATATCCAAG
	reverse	CTTGGATATATAGGTGTCGTAATGGTTCTCCTCCAGCC
**N95T**	*forward*	GGCTGGAGGAGAACCATTACACCACCTATATATCCAAG
	reverse	CTTGGATATATAGGTGGTGTAATGGTTCTCCTCCAGCC
**N95V**	*forward*	GGCTGGAGGAGAACCATTACGTCACCTATATATCCAAG
	reverse	CTTGGATATATAGGTGACGTAATGGTTCTCCTCCAGCC
**S99A**	*forward*	CCATTACAACACCTATATAATCAAGAAGCATGCAGAG
	reverse	CTCTGCATGCTTCTTGATTATATAGGTGTTGTAATGG
**S116R**	*forward*	GGCCTCAAGAAGAATGGGCGCTGCAAACGCGGTCCTCGG
	reverse	CCGAGGACCGCGTTTGCAGCGCCCATTCTTCTTGAGGCC
**L133T**	*forward*	CCAGAAAGCAATCTTGTTTACCCCCCTGCCAGTCTC
	reverse	GAGACTGGCAGGGGGGTAAACAAGATTGCTTTCTGG
**P134A**	*forward*	GCAATCTTGTTTCTCGCCCTGCCAGTCTCTTCTG
	reverse	CAGAAGAGACTGGCAGGGCGAGAAACAAGATTGC
**P134S**	*forward*	GCAATCTTGTTTCTCTCCCTGCCAGTCTCTTCTG
	reverse	CAGAAGAGACTGGCAGGGAGAGAAACAAGATTGC
**L135D**	*forward*	GGCCAGAAAGCAATCTTGTTTCTCCCCGACCCAGTCTCTTCTG
	reverse	CAGAAGAGACTGGGTCGGGGAGAAACAAGATTGCTTTCTGGCC
**L135R**	*forward*	GGCCAGAAAGCAATCTTGTTTCTCCCCCGCCCAGTCTCTTCTG
	reverse	CAGAAGAGACTGGGCGGGGGAGAAACAAGATTGCTTTCTGGCC

Table S5. Scheme of site-directed mutagenesis reactions to obtain FGF1 mutants.

Step	T (°C)	Time (s)
1	95	120
2	95	30
3	T_A	30
4	72	90
5	go back to step 2 and repeat 30 times	
6	72	300
7	12	600

T_A – the annealing temperature of primers, which is given in Table S3.

Table S6. Annealing temperatures of primers in site-directed mutagenesis reactions carried out to obtain FGF1 mutants.

Substitution	T_A (°C)
V51A	66
V54A	65.6
L89A	61.5
L89T	64.1
Y94F	62.4
N95D	61.4
N95T	61
N95V	64.4
S99A	59
S116R	69
L133T	63
P134A	61.4
P134S	61
L135D	65.6
L135R	67.4

T_A – the annealing temperature of primers
